# Causal Relationship Between Cataracts and Gastrointestinal Diseases: A Two-Sample Mendelian Randomization Study

**DOI:** 10.1167/tvst.14.8.27

**Published:** 2025-08-20

**Authors:** Yanchun Li, Qing Min Pan, MengYa Wang, Bin Zhao

**Affiliations:** 1 Department of Ophthalmology, The Second Affiliated Hospital of Shandong First Medical University, Taian, Shandong, People's Republic of China; 2 Department of Colorectal Surgery, The People's Hospital of Feicheng, Feicheng, Shandong, People's Republic of China

**Keywords:** cataract, gastrointestinal diseases, Mendelian randomization, single nucleotide polymorphism, instrumental variable

## Abstract

**Purpose:**

Cataracts, a leading cause of blindness, have been linked to systemic conditions. Previous studies showing associations between cataracts and gastrointestinal diseases have not determined causal relationships. We aimed to investigate the causal relationship between cataracts and 23 gastrointestinal diseases through Mendelian randomization (MR).

**Methods:**

We obtained genome-wide association study (GWAS) datasets for 23 gastrointestinal diseases and cataracts in European populations from the IEU OpenGWAS project. Multivariable MR was performed to account for lipid-related confounders. A two-step MR analysis was conducted to investigate the potential mediating role of 1400 plasma metabolites.

**Results:**

After Bonferroni correction and validation using additional cataract GWAS datasets, gastroesophageal reflux disease (GERD; odds ratio [OR] = 1.131, *P* = 5.85e–5) and celiac disease (OR = 1.012, *P* = 0.002) were found to be associated with increased risks of cataracts. Subgroup analyses revealed that GERD was specifically associated with senile cataract, whereas celiac disease showed suggestive causal relationships with senile cataract, drug-induced cataract, and other cataract types. *RNF5* was downregulated in both celiac disease and lens injury models and was identified as a significant protective factor for senile and other cataracts. Furthermore, the causal effects of GERD and celiac disease on cataracts remained robust after adjusting for lipid profiles, suggesting independence from lipid-related pathways. Notably, 1-linoleoyl-GPE (18:2) was identified as a potential mediator of the causal pathway between GERD and cataract risk (β = 0.008, *P* = 0.035).

**Conclusions:**

These findings reveal causal effects between cataracts and gastrointestinal diseases, providing new insights into their potential biological links.

**Translational Relevance:**

This study revealed a possible association between cataracts and gastrointestinal diseases, supporting the need for targeted screening and early intervention strategies in at-risk populations to improve cataract prevention and comorbidity care.

## Introduction

Cataracts represent a significant contributor to blindness and visual impairment, characterized by clouding of the lens, and they contribute to approximately 2.96% of the global population with vision impairment (65.2 million out of 2.2 billion).^1^^,^^2^ The pathogenesis of cataracts varies across different types. Age-related cataracts, the most common type, are usually associated with aging and oxidative stress.^3^ Genetic factors are important risk factors for various types of cataracts, with heritability estimates varying between 35% and 58%.^4^^,^^5^ Additionally, cataracts have been linked to numerous systemic diseases, including gastrointestinal diseases.^6^

Recent evidence has emphasized the crosstalk between gut health and eye health, known as the gut–eye axis.^7^ This interaction involves the microbiota, the immune system, the nervous system, and nutrient absorption.^8^^,^^9^ However, the relationship between cataracts and the gastrointestinal tract is often overlooked. An evaluation of 272,873 patients with ocular disease showed that cataracts were a more common ocular disease in patients with celiac disease, Crohn's disease, and ulcerative colitis, with prevalence rates of 12%, 22%, and 29.2%, respectively.^10^ Patients with early-onset cataracts have an increased risk of peptic ulcers.^11^ Furthermore, genetic associations between cataracts and gastrointestinal disorders have been observed, indicating that gene expression linked to cataracts is not confined to lens tissues.^5^^,^^12^ Specifically, gene expression in gastrointestinal tissues has shown a significant association with cataracts, with 43 of 202 Bonferroni-significant genes reported in the Genotype-Tissue Expression (GTEx) project.^12^ However, the causal link between cataracts and gastrointestinal diseases is still not well understood.

Increasing studies have applied genome-wide association study (GWAS) and Mendelian randomization (MR) approaches to uncover shared genetic architectures and causal links between ocular diseases and systemic conditions, including neuropsychiatric^13^ and cardiovascular disorders,^14^ highlighting the systemic relevance of eye diseases. In this research, we leveraged the GWAS data to perform the MR analysis to determine if there is a bidirectional causal relationship between 23 gastrointestinal diseases and cataracts. Our study offers novel insights into the potential interplay between gastrointestinal diseases and cataracts.

## Methods

### Study Design

We conducted bidirectional MR analyses to investigate the causality between cataracts and 23 gastrointestinal diseases. The disease included five categories: six upper gastrointestinal diseases, six lower gastrointestinal diseases, six biliary or pancreatic diseases, four liver diseases, and acute appendicitis, as previously described.^15^ Bidirectional MR analysis is conducted to determine the causal direction between two traits (e.g., a certain gastrointestinal disorder and cataract), assessing whether one influences the other or vice versa. For accurate causal inference in MR, single nucleotide polymorphisms (SNPs) utilized as instrumental variables (IVs) must satisfy three principal conditions: (1) a robust association with the exposure is required; (2) they must not be linked to any confounders; and (3) their effect on the outcome should be exclusive via the exposure, with no alternative routes.

### Data Sources

The GWAS datasets were downloaded from the IEU OpenGWAS project (https://gwas.mrcieu.ac.uk). Specific information on the GWAS datasets is presented in Supplementary Table S1. GWAS summary data for the included gastrointestinal traits were obtained from three major sources (Supplementary Table S2): (1) the GWAS Catalog (ebi-a-), which includes meta-analyzed summary statistics from international consortia using clinically confirmed cases and population-matched controls; (2) UK Biobank–based GWAS (ukb-a-/ukb-d-), where cases and controls were defined using International Classification of Diseases (ICD)-coded diagnoses and self-reported data, harmonized via the PheCODE framework; and (3) FinnGen (finn-b-), where cases were identified using ICD-coded diagnoses from national health registers, and controls were individuals free of the relevant codes. The IEU OpenGWAS project performs additional filtering and harmonization when integrating GWAS summary statistics from various sources. The summary statistics of 1400 plasma metabolites were obtained from the GWAS catalog (https://www.ebi.ac.uk/gwas/summary-statistics).

The datasets GSE112102 (spike biopsy samples from 12 patients with celiac disease and 12 control samples; GPL10558), GSE102991 (intestinal epithelial cell samples from biopsies of four patients with celiac disease and four clinical controls; GPL6883), and GSE213546 (four human lens and four age-related cataract; GPL10558) were obtained from the Gene Expression Omnibus database (https://www.ncbi.nlm.nih.gov/geo/). Bulk RNA sequencing data from 52 lens epithelial cell samples, collected at six time points over a 0- to 120-hour period following simulated cataract surgery, were obtained from the Lens Injury Response Time Series (LIRTS) Viewer (https://lirts.dbi.udel.edu/Home).

### Screening of IVs

SNPs were acquired from GWAS datasets, including details such as effect allele, effect size (beta), standard error, and *P* value. SNPs were selected as IVs for exposure factors with a criterion of *P* < 5.0 × 10^−8^. Due to the limited number of IVs obtained for the 13 gastrointestinal diseases, there may be insufficient statistical power. Therefore, relaxing the threshold to *P* < 5 × 10^−6^ could help capture more genetic signals and enhance statistical power while controlling for false positives.^16^^,^^17^ These diseases included esophageal cancer, duodenal ulcer, gastric ulcer, acute gastritis, chronic gastritis, Crohn's disease, diverticular disease, acute pancreatitis, chronic pancreatitis, pancreatic cancer, nonalcoholic fatty liver disease, alcoholic liver disease, and cirrhosis (Supplementary Table S1). The criterion of plasma metabolites was *P* < 1.0 × 10^−5^.

To minimize linkage disequilibrium (LD) bias, SNPs associated with the exposure were selected with an LD threshold of *R*^2^ < 0.001 and a genetic distance of 10,000 kb. The strength of the IVs was assessed using the *F*-statistic, calculated as *F* = (β_exposure_/SE_exposure_)2. β_exposure_ and SE_exposure_ represent the effect value and standard error of the exposure dataset, respectively. *F* > 10 indicates no weak IV bias.^18^

### Statistical Analysis

Summary statistics from exposure and outcome datasets were harmonized to ensure that SNP effects on exposure and outcome corresponded to the same alleles. Several methods were employed in the bidirectional two-sample MR analysis, including inverse variance weighting (IVW), MR–Egger regression, weighted median, simple mode, and weighted mode. The primary method we employed was IVW, which combines Wald ratio estimates from SNPs that satisfy the IV assumptions, providing a consistent estimate of the causal effect of exposure on outcome. The IVW method yields the most reliable results when horizontal pleiotropy is absent.^19^^,^^20^ The weighted median method offers a consistent estimate of the causal effect when more than half of the SNPs are valid IVs.^21^ MR–Egger regression, which tests for horizontal pleiotropy, could provide an unbiased causal estimate even when such pleiotropy is present.^22^ The accuracy of the results is improved with the weighted median method compared to the MR–Egger method.^23^ Simple mode and weighted mode analyses were conducted as supplementary analyses.^24^ The Mendelian randomization pleiotropy residual sum and outlier (MR-PRESSO) test was used to identify and adjust for horizontal pleiotropy by excluding outliers.^25^ The TwoSampleMR^26^ and MR-PRESSO^27^ packages in R (R Foundation for Statistical Computing, Vienna, Austria) were used to perform statistical analyses, with a significance threshold set at α = 0.05 (*P* < 0.05). To address multiple testing, we applied Bonferroni correction, factoring in the total number of exposures and outcomes.^28^ A threshold of *P* < 0.0022 (0.05/23) was used to indicate robust statistical significance for the MR analyses involving 23 gastrointestinal diseases and cataracts, whereas results significant at *P* = 0.0022 to 0.05 after correction were classified as potential associations. For subgroup analyses involving three cataract subtypes and five gastrointestinal diseases (15 tests), the threshold was set at 0.0033. For replication analyses using two cataract datasets and five gastrointestinal diseases (10 tests), the threshold was 0.005.

### Heterogeneity and Sensitivity Test

IVW and MR-Egger regression were used for heterogeneity tests among IVs. The heterogeneity was quantified using Cochran's *Q*-test. *P* < 0.05 suggests the presence of heterogeneity, so the random effects IVW model was employed. The leave-one-out method was employed to determine whether any single SNP had a significant impact on the MR results.

### Multivariable Mendelian Randomization and Mediation Analysis

The multivariable Mendelian randomization (MVMR) method was used to adjust for the influence of lipid metabolism–related indicators, including high-density lipoprotein (HDL) cholesterol, low-density lipoprotein (LDL) cholesterol, total cholesterol, and triglycerides (TGs). We performed a two-step MR analysis to investigate whether plasma metabolites mediate the causal effect of gastrointestinal diseases on cataract risk. In the first step, the causal effects of 1400 plasma metabolites on cataracts were evaluated. In the second step, the causal effects of gastrointestinal diseases on plasma metabolites were assessed. Finally, the product of coefficients method was applied to estimate the mediated effect, and the delta method was used to calculate the standard errors for the mediated effects.

## Results

### Causal Effects of Gastrointestinal Diseases on Cataracts

Gastrointestinal diseases were the exposure factors and cataract was the outcome variable. Following the selection of SNPs based on the aforementioned criteria, we excluded palindromic SNPs (A/T or G/C) and those unavailable in the outcome data. Consequently, we identified between four and 66 SNPs as IVs for 23 gastrointestinal diseases. The *F*-statistics for all identified IVs exceeded 10.

**Figure 1.**
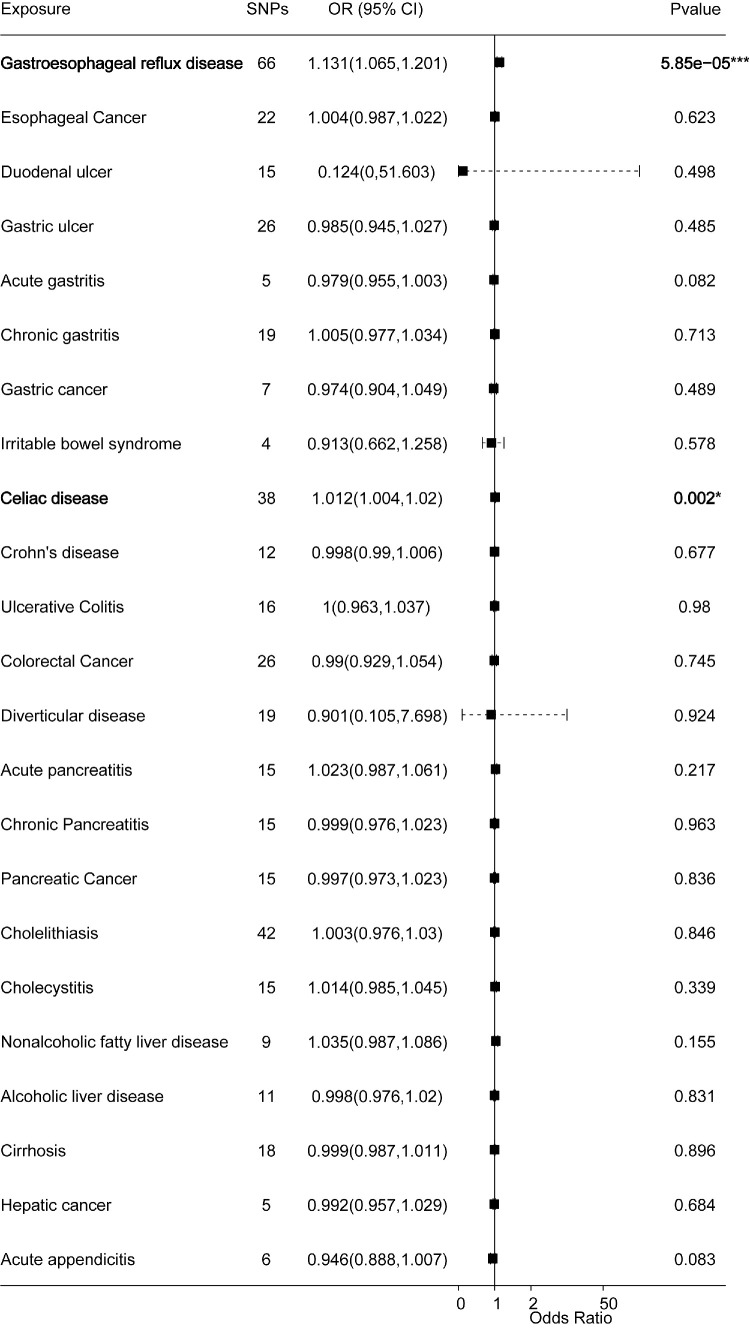
Forest plots of the causal effect of 23 gastrointestinal diseases on cataracts (IVW method results).

MR analyses support the causal links between genetic susceptibility to gastroesophageal reflux disease (GERD) and celiac disease and an increased risk of cataracts. The IVW model showed a significant causation between GERD and cataracts (odds ratio [OR] = 1.131; 95% confidence interval [CI], 1.065–1.201; *P* = 5.85E–05), as well as between celiac disease and cataracts (OR = 1.012; 95% CI, 1.004–1.02; *P* = 0.0023) (Fig. 1, Supplementary Table S3). The intercepts of the MR–Egger regression were near 0, suggesting no horizontal pleiotropy of IVs in gastrointestinal diseases and indicating a negligible likelihood of affecting the MR results (Supplementary Table S4).

**Figure 2.**
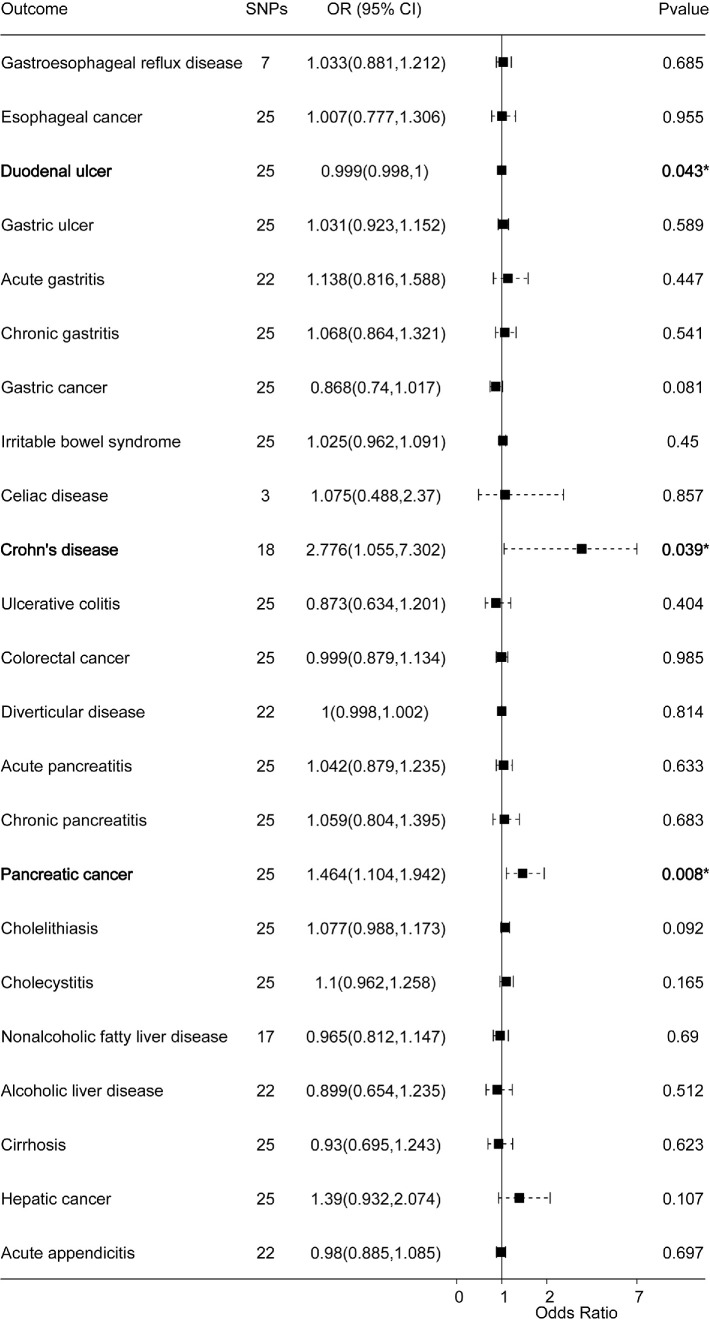
Forest plots of the causal effect of cataracts on 23 gastrointestinal diseases (IVW method results).

Both MR–Egger and IVW analyses for GERD and celiac disease showed no heterogeneity (Supplementary Table S5); however, heterogeneity was found between nine gastrointestinal diseases and cataracts (Supplementary Table S6). Therefore, random effects IVW and MR-PRESSO tests were applied, which indicated no causal effect between these diseases and cataract risk (Supplementary Table S7). The results of the leave-one-out method indicated that the causal effect between GERD and celiac disease and an increased risk of cataracts was robust and not influenced by any single SNP (Supplementary Figs. S1A–S1B). The direction of MR–Egger was inconsistent compared to other MR analysis methods for GERD (Figs. 2C, 2D). However, the MR–Egger results were nonsignificant with a 95% CI including 1. Because the IVW method is more reliable under the assumption of no horizontal pleiotropy, a potential causal effect of GERD on cataract risk cannot be excluded.

### Causal Effects of Cataracts on Gastrointestinal Diseases

Cataract was the exposure factor and gastrointestinal diseases were the outcome variable. We identified between three and 25 SNPs as IVs for cataracts. All identified IVs had *F*-statistics exceeding 10. MR analyses revealed that cataracts were linked to a heightened risk of Crohn's disease (IVW result: OR = 2.776; 95% CI, 1.055–7.302; *P* = 0.039) and pancreatic cancer (IVW result: OR = 1.464; 95% CI, 1.104–1.942; *P* = 0.008) (Fig. 2, Supplementary Table S7). Meanwhile, cataracts were related to the risk of duodenal ulcers (IVW result: OR = 0.9988; 95% CI, 0.9977–0.9999; *P* = 0.043). Furthermore, there was no horizontal pleiotropy of IVs (Supplementary Table S8). However, heterogeneity was found between cataracts and six gastrointestinal diseases (Supplementary Table S9). Subsequently, random-effects IVW and MR-PRESSO tests were applied, which indicated no causal effect between cataracts and the risks of these diseases (Supplementary Table S6). The leave-one-out method indicated the robustness of the MR analysis between cataracts and duodenal ulcers, whereas the causal effects of cataracts on Crohn's disease and pancreatic cancer may be driven by single SNPs (Supplementary Fig. S2). Therefore, these causations should be interpreted with caution. Notably, Bonferroni correction (*P* < 0.0022) indicated that these causal relationships were suggestive.

### Subgroup Analysis of Cataracts

**Figure 3.**
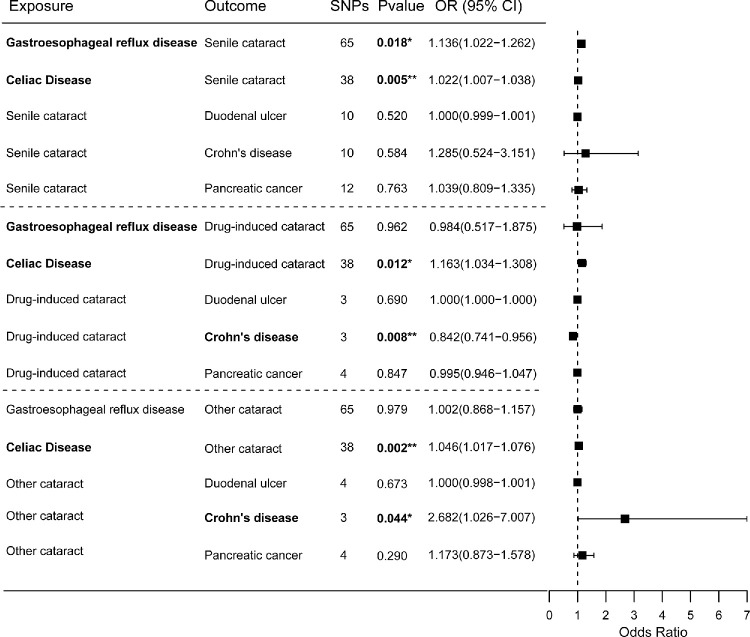
Forest plots of the causal analysis between cataract subgroups and five gastrointestinal diseases (IVW method results).

To further explore the specificity of the causal relationships, we conducted subgroup MR analyses by three cataract subtypes (senile cataract, drug-induced cataract, and other cataract) (Fig. 3). The results showed that a suggestive causality between GERD and senile cataract risk (IVW result: OR = 1.136; 95% CI, 1.022–1.262; *P* = 0.018). Furthermore, celiac disease was found to have suggestive causations with senile cataract (IVW result: OR = 1.022; 95% CI, 1.007–1.038; *P* = 0.005), drug-induced cataract (IVW result: OR = 1.163; 95% CI, 1.034–1.308; *P* = 0.012), and other cataract (IVW result: OR = 1.046; 95% CI, 1.017–1.076; *P* = 0.002). No horizontal pleiotropy was found. Although heterogeneity was observed in the MR analysis between celiac disease and other cataracts (Cochran's *Q*-test: IVW, *P* = 0.020; MR–Egger, *P* = 0.024), no significant outliers were detected by MR-PRESSO. The random-effects IVW model still indicated a potential causal association (OR = 1.046; 95% CI, 1.017–1.076; *P* = 0.002). Additionally, leave-one-out analysis suggested that no single SNP had a disproportionate influence on these causal associations (Supplementary Figs. S3–S5).

These results also revealed the potential causal effects of drug-induced cataract (IVW result: OR = 0.842; 95% CI, 0.741–0.956; *P* = 0.008) and other cataract (IVW result: OR = 2.682; 95% CI, 1.026–7.007; *P* = 0.044) on Crohn's disease. However, leave-one-out analysis suggested that these associations might be influenced by individual SNPs (Supplementary Figs. S4, S5).

### Potential Role of RNF5 Downregulation in Linking Celiac Disease to Cataract Risk

**Figure 4.**
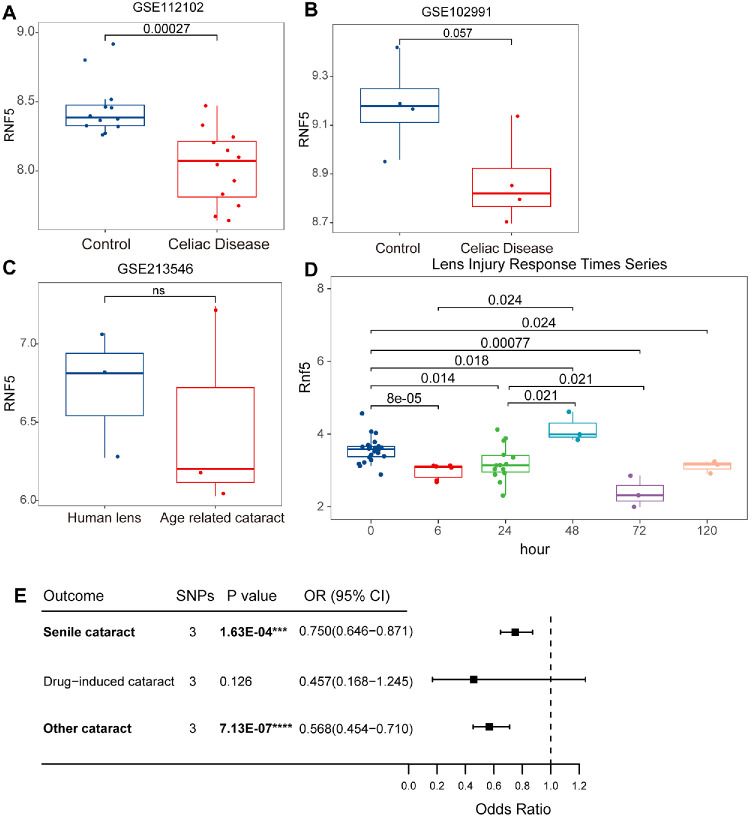
The potential role of *RNF5* in celiac disease and cataract. (**A**) Box plot shows the expression levels of *RNF5* in celiac disease and control samples from the GSE112102 dataset. (**B**) Box plot shows the expression levels of *RNF5* in celiac disease and control samples from the GSE102991 dataset. (**C**) Box plot shows the expression levels of *RNF5* in human lens and lens from age-related cataract from the GSE112102 dataset. (**D**) Box plot shows *RNF5* expression levels at different time points in the lens injury model from LIRTS. (**E**) Forest plots of the causal effects of *RNF5* on three cataract subtypes (IVW method results).

We identified an instrumental SNP for celiac disease, rs2269423, that showed a significant association with both age-related cataract (*P* = 0.003) and other cataract (*P* = 2.03798e–05). According to dbSNP annotations, rs2269423 is located within 2 kb upstream of ring finger protein 5 (RNF5). RNF5 was downregulated in celiac disease compared to controls (Figs. 4A, 4B). Notably, RNF5 also showed a decreasing trend in age-related cataract compared to healthy human lens tissue (Fig. 4C). In a mouse model of lens epithelial injury (LIRTS), RNF5 expression was significantly reduced at all time points except at 48 hours (Fig. 4D). Furthermore, MR analysis indicated that RNF5 is a significant protective factor for both senile and other cataracts (Fig. 4E). No horizontal pleiotropy or heterogeneity was detected, and the robustness of the causal estimates was supported by leave-one-out sensitivity analysis (Supplementary Fig. S6). These findings suggest that RNF5 may serve as a potential molecular link between celiac disease and cataract risk.

### Validation of MR Results Across Independent Datasets

**Figure 5.**
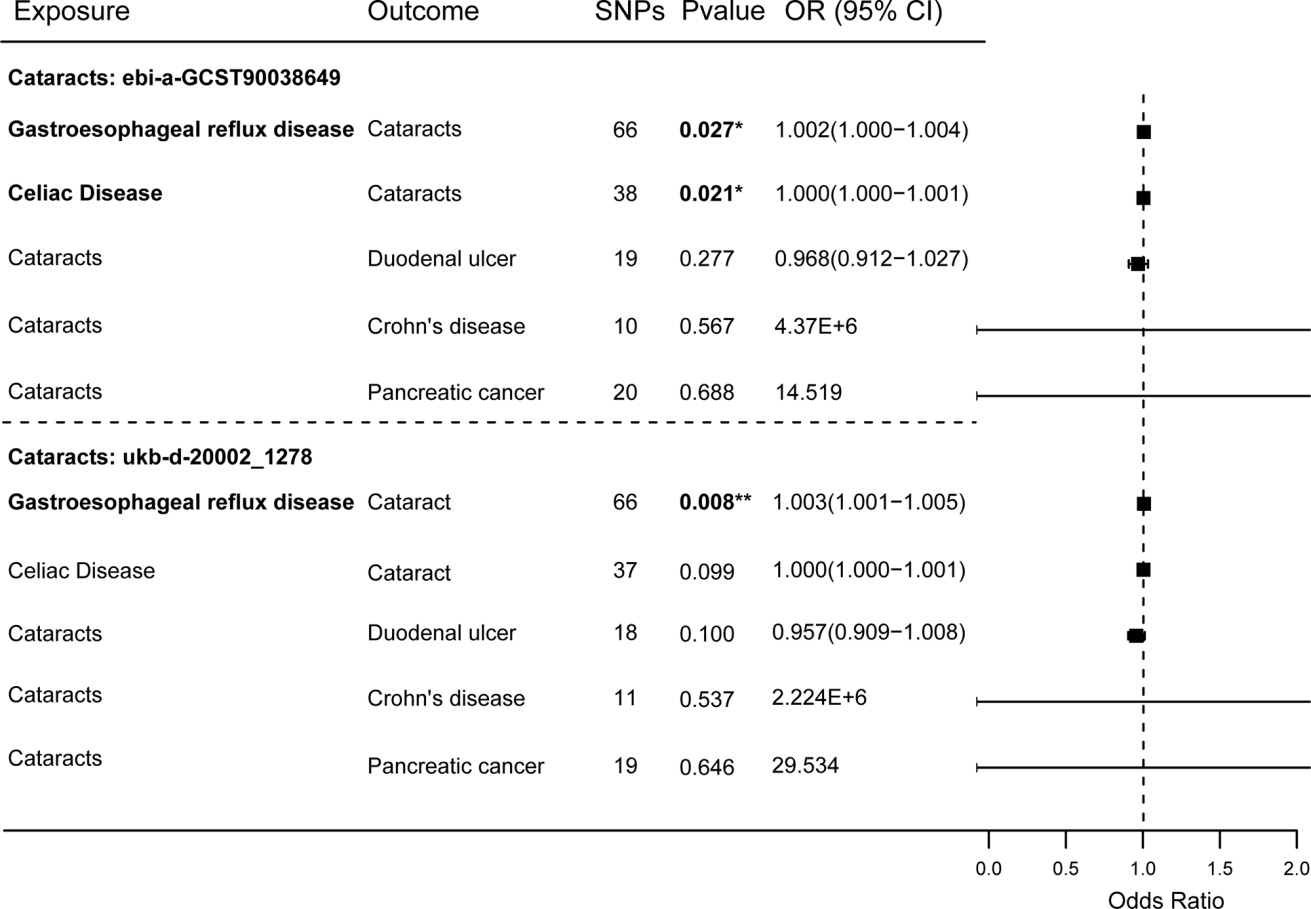
Forest plots of the causal analysis between cataract subgroups and five gastrointestinal diseases validated using different cataract datasets.

We included additional GWAS summary data of cataracts to validate the MR results (Fig. 5, Supplementary Figs. S7–S8). Both datasets suggest a potential causal relationship, indicating that GERD may increase the risk of cataracts (Fig. 5), and leave-one-out analyses confirmed the robustness of this causation (Supplementary Figs. S7A, S8A). One dataset also showed a suggestive causal effect of celiac disease on cataract risk; however, this effect may be influenced by two specific SNPs (Fig. 5, Supplementary Fig. S7B).

### MVMR and Analysis of the Potential Mediating Role of Plasma Metabolites

We conducted MVMR to assess whether the potential causal effects of GERD and celiac disease on cataract risk were confounded by lipid metabolism–related traits (Supplementary Table S10). After adjusting for genetically predicted levels of HDL, LDL, and TGs, both GERD and celiac disease remained significantly associated with cataract risk, suggesting that their effects may be independent of these lipid-related factors.

**Figure 6.**
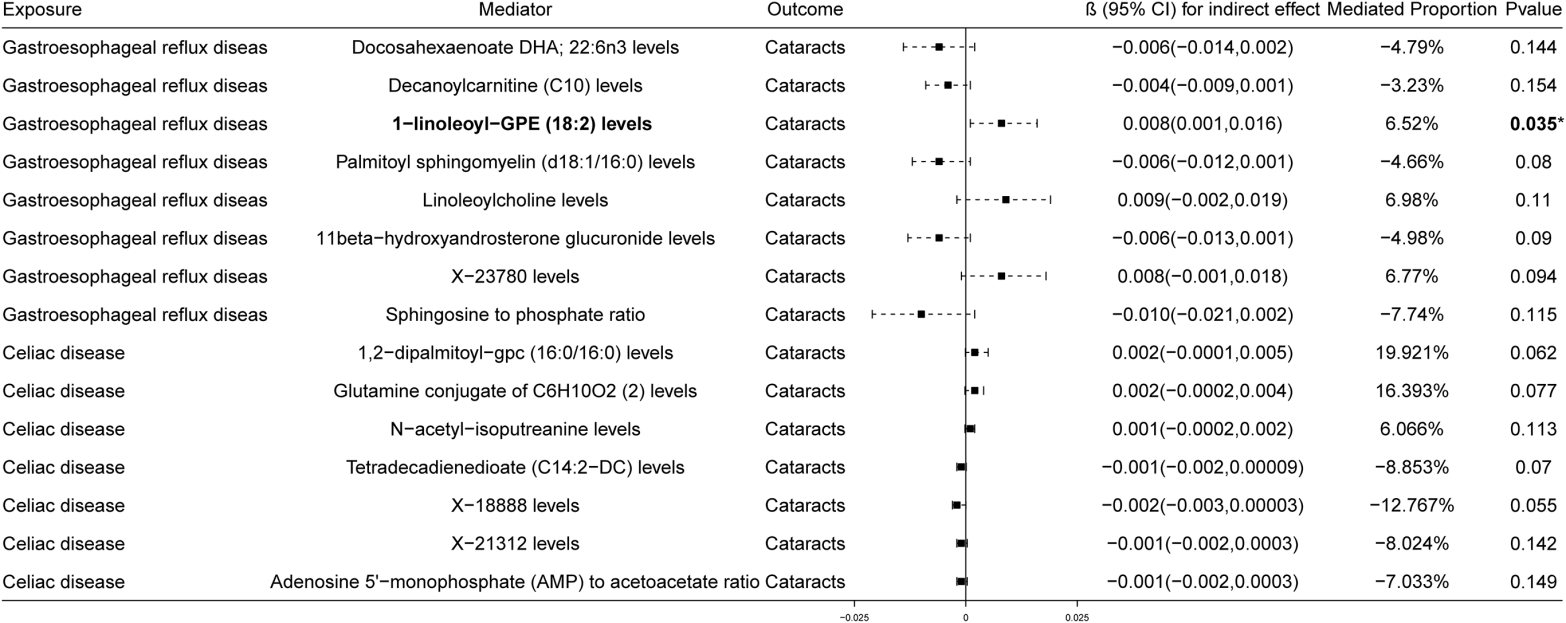
Mediation analysis of plasma metabolites as potential mediators of the causal effects of gastroesophageal reflux disease and celiac disease on cataract risk.

We performed a two-step MR analysis to investigate whether plasma metabolites may mediate the causal effects of GERD and celiac disease on cataract risk. In total, 67 plasma metabolites were found to have suggestive causal associations with cataracts, including 30 metabolites potentially increasing the risk and 37 potentially reducing it (Supplementary Table S11). GERD may be associated with altered levels of eight plasma metabolites (Supplementary Table S12). Mediation analysis showed that these metabolites collectively accounted for a total effect size of 0.123 in the causal pathway from GERD to cataracts. Among them, 1-linoleoyl-GPE (18:2) levels may mediate the causal relationship between GERD and cataracts (β = 0.008; 95% CI, 0.001–0.016; *P* = 0.035), with an estimated mediation proportion of 6.52% of the total effect (Fig. 6). Celiac disease was associated with changes in levels of seven plasma metabolites (Supplementary Table S13). These metabolites collectively contributed to a total mediated effect of 0.012. However, none of the mediation effects reached statistical significance (*P* > 0.05). These results suggest that 1-linoleoyl-GPE (18:2) levels may mediate the causal effect of GERD on increased cataract risk, whereas plasma metabolites appear to have no significant mediating role in the causal pathway from celiac disease to cataract.

## Discussion

Gastrointestinal diseases often present with extraintestinal manifestations, including various ocular features.^29^ Cataracts, a common ocular condition, are frequently associated with systemic diseases.^6^ In this study, we identified suggestive causal relationships between cataracts and five gastrointestinal diseases, providing genetic evidence for their association. GERD may affect organs adjacent to or distant from the esophagus because they lack protective mechanisms similar to those of the esophagus.^30^ A retrospective analysis found that up to 19% of patients undergoing cataract surgery under local anesthesia also had GERD.^31^ In addition, a prospective cohort study evaluated the relationship between GERD and its comorbidities and the incidence of cataracts during a 1-year follow-up period.^32^ The results showed that various manifestations of GERD, including Barrett's esophagus, esophagitis, and simple reflux (hazard ratio [HR] = 1.40; 95% CI, 1.08–1.81) were associated with an increased risk of cataracts. Although the MR–Egger analysis showed some inconsistency, this may be due to its susceptibility to weak instrument bias and directional pleiotropy.^33^ In contrast, the IVW method produced more robust results under the assumption of no horizontal pleiotropy. The replication dataset produced consistent results, enhancing a causal link between GERD and cataract risk.

Further subgroup analysis revealed a suggestive causal relationship between GERD and senile cataracts specifically. We hypothesize two possible underlying mechanisms: oxidative stress and inflammation. Protein aggregation and degradation in lens fibers, largely driven by oxidative damage, are known hallmarks of senile cataract formation.^34^^,^^35^ In GERD, gastric acid reflux damages the esophageal mucosa and relaxes the esophageal sphincter, which causes oxidative damage.^36^ A systemic decrease in antioxidant enzyme levels has been observed in patients with laryngopharyngeal reflux (LPR, the extraesophageal manifestation of GERD).^37^ Pepsin, a digestive enzyme frequently refluxed in LPR, can induce proinflammatory cytokines and receptor expression in upper airway epithelial cells.^38^ Notably, pepsin has been detected in the tears of 51% of LPR patients, along with elevated HLA-DR expression, suggesting an immune-activating effect on the ocular surface.^39^ Given that immune dysregulation and chronic inflammation contribute to the progression of senile cataracts, it is plausible that GERD-related oxidative and inflammatory changes may underlie this causal relationship.

Celiac disease is an autoimmune disease of the small intestine. Previous cases have reported bilateral cataracts as a presenting feature of celiac disease.^40^ In a cohort study of 28,756 patients with biopsy-proven celiac disease, there was an increased risk of cataracts (HR = 1.8; 95% CI, 1.19–1.36). This association may be related to immune response, nutrient absorption, and oxidative stress.^41^ Our MR analysis supports this causation, which is consistent with previous research.^42^ Furthermore, this causation may potentially involve multiple subtypes of cataract. Celiac disease patients often exhibit reduced antioxidant capacity, which can lead to oxidative stress,^43^ thereby exacerbating glutathione depletion and lipid peroxidation in the crystalline lens.^34^ Dysbiosis in the gut microbiome may also contribute,^44^ as intestinal metabolites such as tauroursodeoxycholic acid have been shown to effectively reduce the apoptosis of lens epithelial cells.^45^ Furthermore, chronic malabsorption in celiac disease can lead to deficiencies in zinc, selenium, and vitamins C and E, all of which are critical for lens health.^46^ The use of systemic corticosteroids requires caution in patients with celiac disease, as prolonged use may lead to posterior subcapsular cataract formation.^47^

A retrospective study has reported that gastric bypass surgery may reduce the risk of cataracts.^48^ This appears to contrast with our findings. The surgery likely reduces cataract risk by improving obesity and related metabolic disturbances, which are known risk factors for cataract development.^49^ Although preliminary analyses suggested potential associations between cataract and Crohn's disease, pancreatic cancer, and duodenal ulcer, leave-one-out analysis indicated that these results may not be robust. Previous studies have suggested possible shared genetic factors among these diseases, such as nucleotide-binding oligomerization domain-containing protein 2,^50^^,^^51^ glutathione peroxidase 1 polymorphism (rs1800668),^52^^,^^53^ and breast cancer type 2.^54^ However, to date, there is no epidemiological or genetic evidence supporting a causal relationship between these gastrointestinal diseases and cataract.

We identified a SNP (rs2269423) that may play a critical role in the causal pathway from celiac disease to cataract. Its corresponding gene, *RNF5*, is an E3 ubiquitin ligase involved in regulating the stability and function of various proteins through ubiquitination. It plays important roles in antiviral immunity, cell-cycle regulation, and tumorigenesis.^55^^,^^56^ A family member, *RNF114*, has been shown to participate in lens protein turnover and homeostasis.^57^ Currently, no studies have directly investigated the role of *RNF5* in either celiac disease or cataract. Our findings indicate that *RNF5* tends to be downregulated in lens injury models and functions as a protective factor against cataracts, suggesting its potential role in maintaining lens epithelial integrity. We hypothesize that the downregulation of *RNF5* in celiac disease may result in the loss of this protective effect, thereby increasing susceptibility to cataract. However, further studies are necessary to confirm the role of *RNF5* in both diseases and to assess its expression in the lenses of patients with celiac disease.

Previous studies have revealed a causal association between plasma metabolites and cataract, identifying 13-HODE, 9-HODE, docosadienoate, and 2-naphthol sulfate as key metabolites.^58^ Our results further support this by uncovering causal relationships between 67 plasma metabolites and cataract risk, providing additional genetic evidence. Moreover, we found that 1-linoleoyl-GPE (18:2) may mediate the causal effect of GERD on cataract risk. 1-Linoleoyl-GPE (18:2) is a lysophospholipid that has been identified as a protective or pathogenic metabolite in various diseases and may be associated with metabolic and immune-related characteristics.^59^^,^^60^ Additionally, our MVMR analysis, adjusting for key blood lipid traits, suggested that GERD may have an independent causal effect on cataract risk, likely through pathways beyond conventional lipid metabolism.

This study has several limitations. First, some individuals classified as controls may have had undiagnosed early-stage lens opacities or may have gone on to develop cataracts later in life. This non-differential misclassification, particularly relevant in age-progressive conditions such as cataract, may bias the MR estimates toward the null. Although the confounding factors and statistical efficiency of the two-sample MR analysis were good, inconsistencies in the quality and source of the data may introduce systematic bias. Second, the study was limited to a European population, which affects the applicability of the results to other populations. Different ethnic groups may have distinct genetic backgrounds and environmental exposures, which could influence the observed associations. Third, although relaxing the SNP selection threshold to *P* < 5 × 10^−6^ is a result of balancing statistical bias and power, it may still lead to the introduction of potentially non-informative tools and increase the risk of pleiotropy, which could bias the results. Although we tested for pleiotropy, it is challenging to completely rule it out. Forth, some gastrointestinal disease datasets and the cataract dataset were derived from the same biobank, which may introduce partial participant overlap and potential bias in the MR estimates. To reduce this risk, we performed subgroup analyses and validation using datasets from different, independent sources whenever possible. Finally, the observed statistical power and effect sizes were relatively modest, which may limit the immediate clinical applicability of our findings. However, the consistent directionality and biological plausibility of these associations suggest potential translational value. Patients with GERD and celiac disease may benefit from closer ophthalmologic monitoring, especially as they age or present other risk factors for cataract development. Due to the relatively small effect sizes and the complexity of cataract pathogenesis, larger prospective studies and mechanistic research are necessary to validate these associations and to evaluate their practical utility in clinical risk assessment and management.

## Conclusions

To our knowledge, our study is the first to examine the causal links between cataracts and gastrointestinal diseases using MR. The findings provide genetic evidence for potential associations among GERD, celiac disease, and increased cataract risk. Further research, especially with more homogeneous populations, and prospective studies are necessary to confirm these associations and clarify the underlying mechanisms.

## Supplementary Material

Supplement 1

Supplement 2
