# Supplementary material for: Causal Relationship Between Cataracts and Gastrointestinal Diseases: A Two-Sample Mendelian Randomization Study
Source: Transl Vis Sci Technol. 2025 Aug 20;14(8):27. doi: 10.1167/tvst.14.8.27 (PMC12372943; doi:10.1167/tvst.14.8.27)
Supplement: Supplement 1 [file tvst-14-8-27_s001.docx]

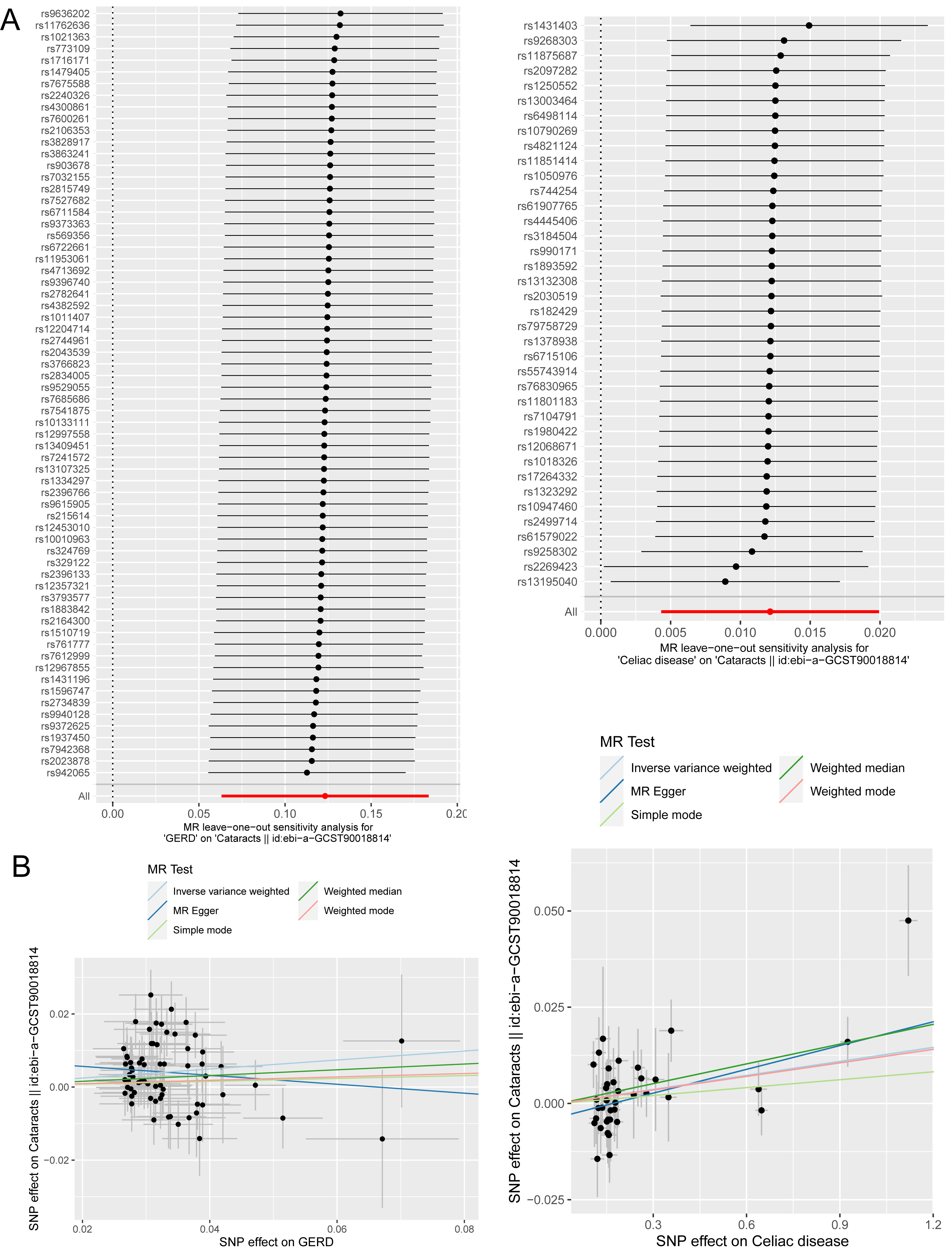


**Figure S1. The causal effect of gastrointestinal diseases on cataracts.** (A) Leave-one-out plots. (B) Scatter plots. Gastroesophageal reflux disease (left). Celiac disease (right).


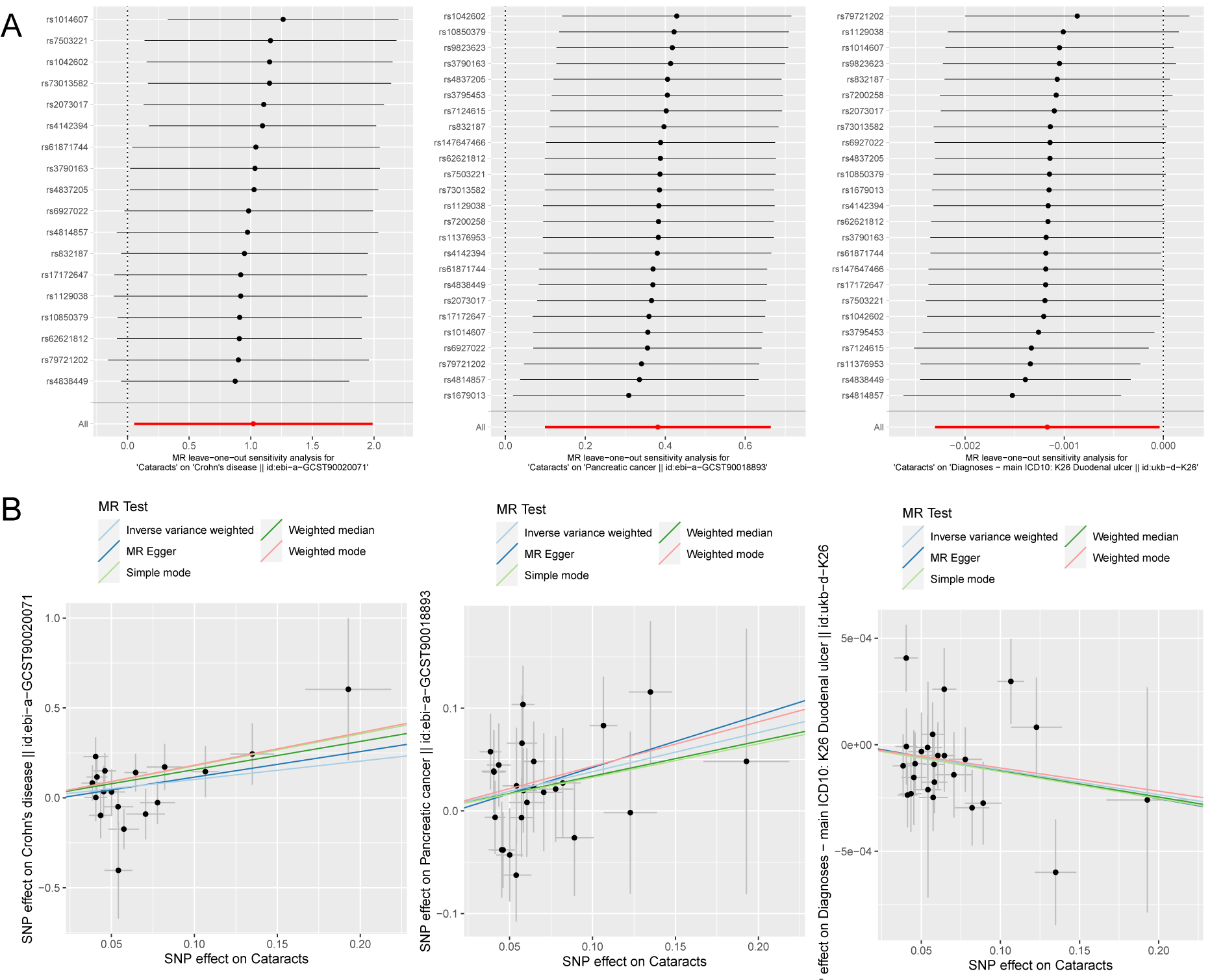


**Figure S2. The causal effect of cataracts on gastrointestinal diseases.** (A) Leave-one-out plots. (B) Scatter plots. Crohn's disease (left). Pancreatic cancer (middle). Duodenal ulcers (right).


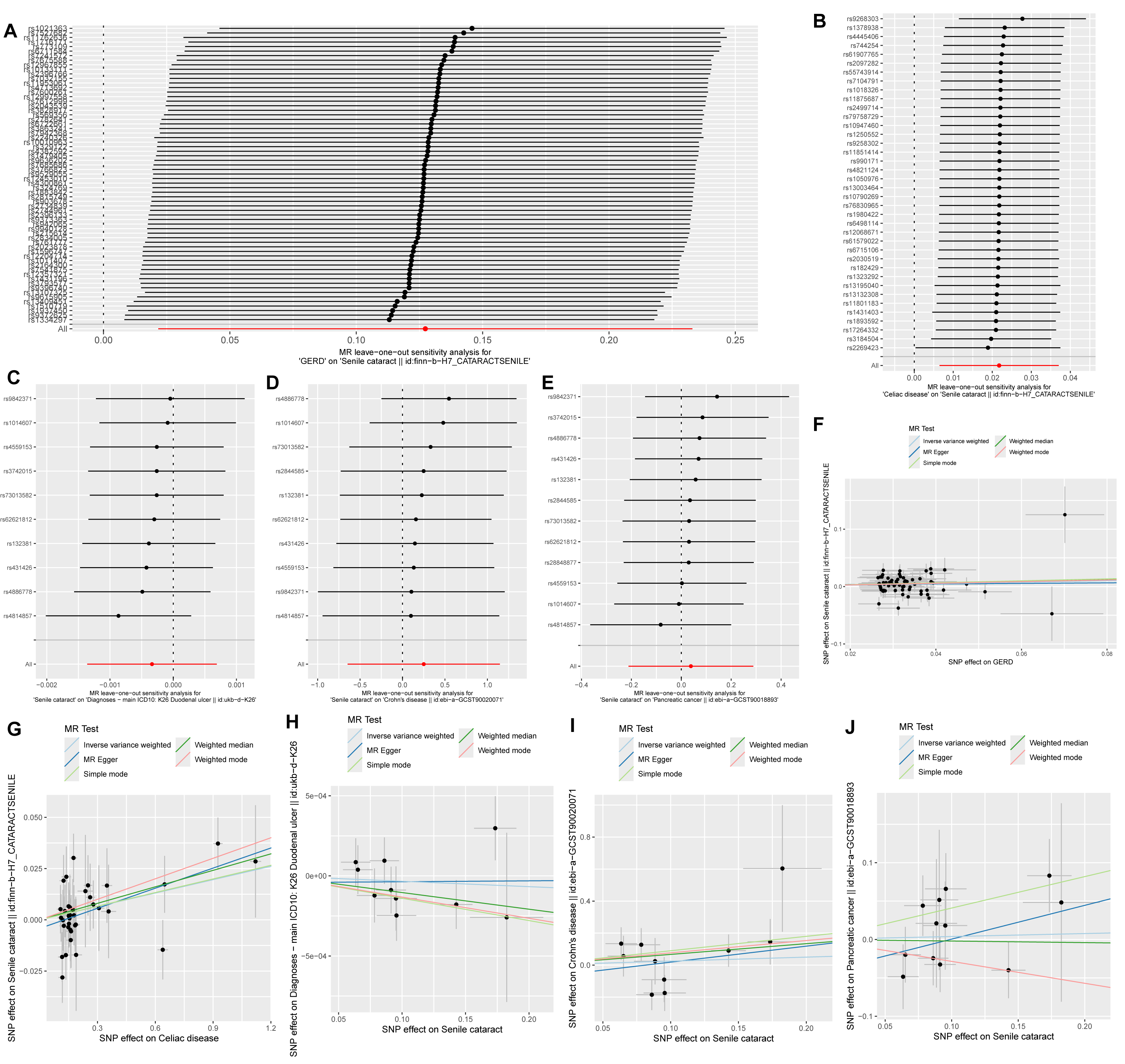


**Figure S3. The causal analysis between senile cataract and gastrointestinal diseases.** (A) Leave-one-out plots for the causal effect of gastroesophageal reflux disease (GERD) on senile cataract. (B) Leave-one-out plots for the causal effect of celiac disease on senile cataract. (C) Leave-one-out plots for the causal effect of senile cataract on duodenal ulcers. (D) Leave-one-out plots for the causal effect of senile cataract on Crohn's disease. (E) Leave-one-out plots for the causal effect of senile cataract on pancreatic cancer. (F) Scatter plots for the causal effect of GERD on senile cataract. (G) Scatter plots for the causal effect of celiac disease on senile cataract. (H) Scatter plots for the causal effect of senile cataract on duodenal ulcers. (I) Scatter plots for the causal effect of senile cataract on Crohn's disease. (J) Scatter plots for the causal effect of senile cataract on pancreatic cancer.


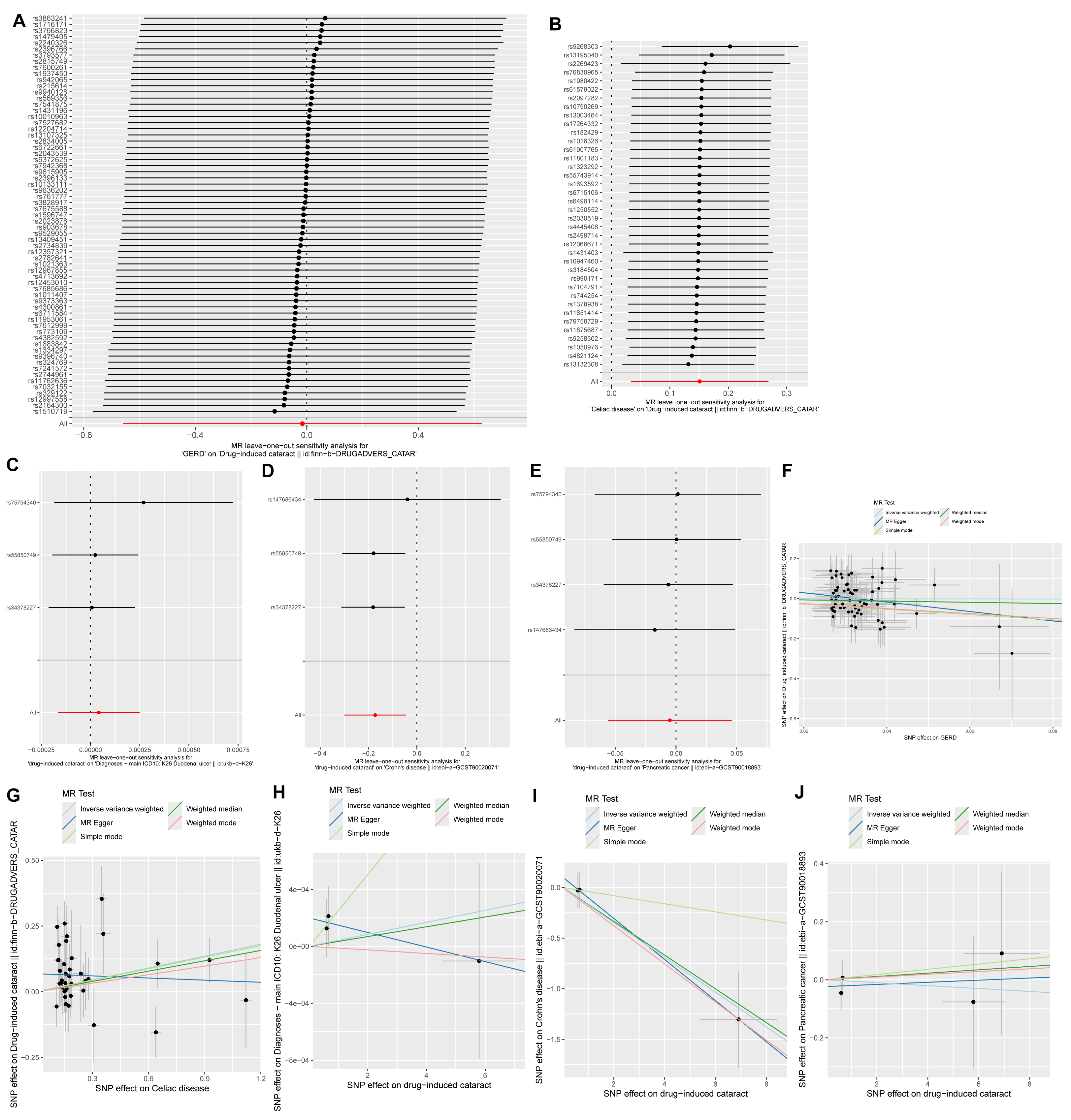


**Figure S4. The causal analysis between** **drug-induced cataract and gastrointestinal diseases.** (A) Leave-one-out plots for the causal effect of gastroesophageal reflux disease (GERD) on drug-induced cataract. (B) Leave-one-out plots for the causal effect of celiac disease on drug-induced cataract. (C) Leave-one-out plots for the causal effect of drug-induced cataract on duodenal ulcers. (D) Leave-one-out plots for the causal effect of drug-induced cataract on Crohn's disease. (E) Leave-one-out plots for the causal effect of drug-induced cataract on pancreatic cancer. (F) Scatter plots for the causal effect of GERD on drug-induced cataract. (G) Scatter plots for the causal effect of celiac disease on drug-induced cataract. (H) Scatter plots for the causal effect of drug-induced cataract on duodenal ulcers. (I) Scatter plots for the causal effect of drug-induced cataract on Crohn's disease. (J) Scatter plots for the causal effect of drug-induced cataract on pancreatic cancer.


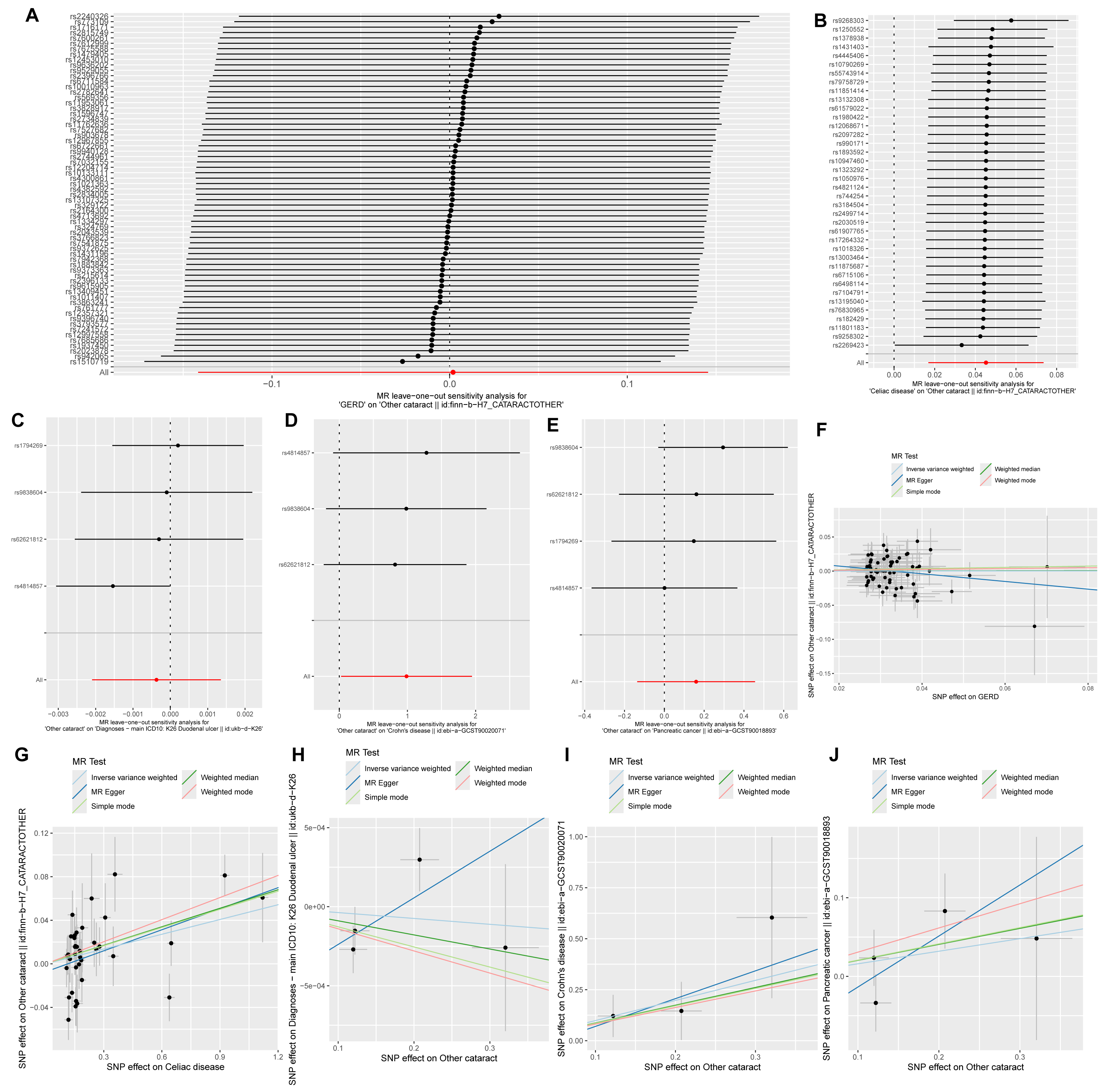


**Figure S5. The causal analysis of other cataract and gastrointestinal diseases.** (A) Leave-one-out plots for the causal effect of gastroesophageal reflux disease (GERD) on other cataract. (B) Leave-one-out plots for the causal effect of celiac disease on other cataract. (C) Leave-one-out plots for the causal effect of other cataract on duodenal ulcers. (D) Leave-one-out plots for the causal effect of other cataract on Crohn's disease. (E) Leave-one-out plots for the causal effect of other cataract on pancreatic cancer. (F) Scatter plots for the causal effect of GERD on other cataract. (G) Scatter plots for the causal effect of celiac disease on other cataract. (H) Scatter plots for the causal effect of other cataract on duodenal ulcers. (I) Scatter plots for the causal effect of other cataract on Crohn's disease. (J) Scatter plots for the causal effect of other cataract on pancreatic cancer.


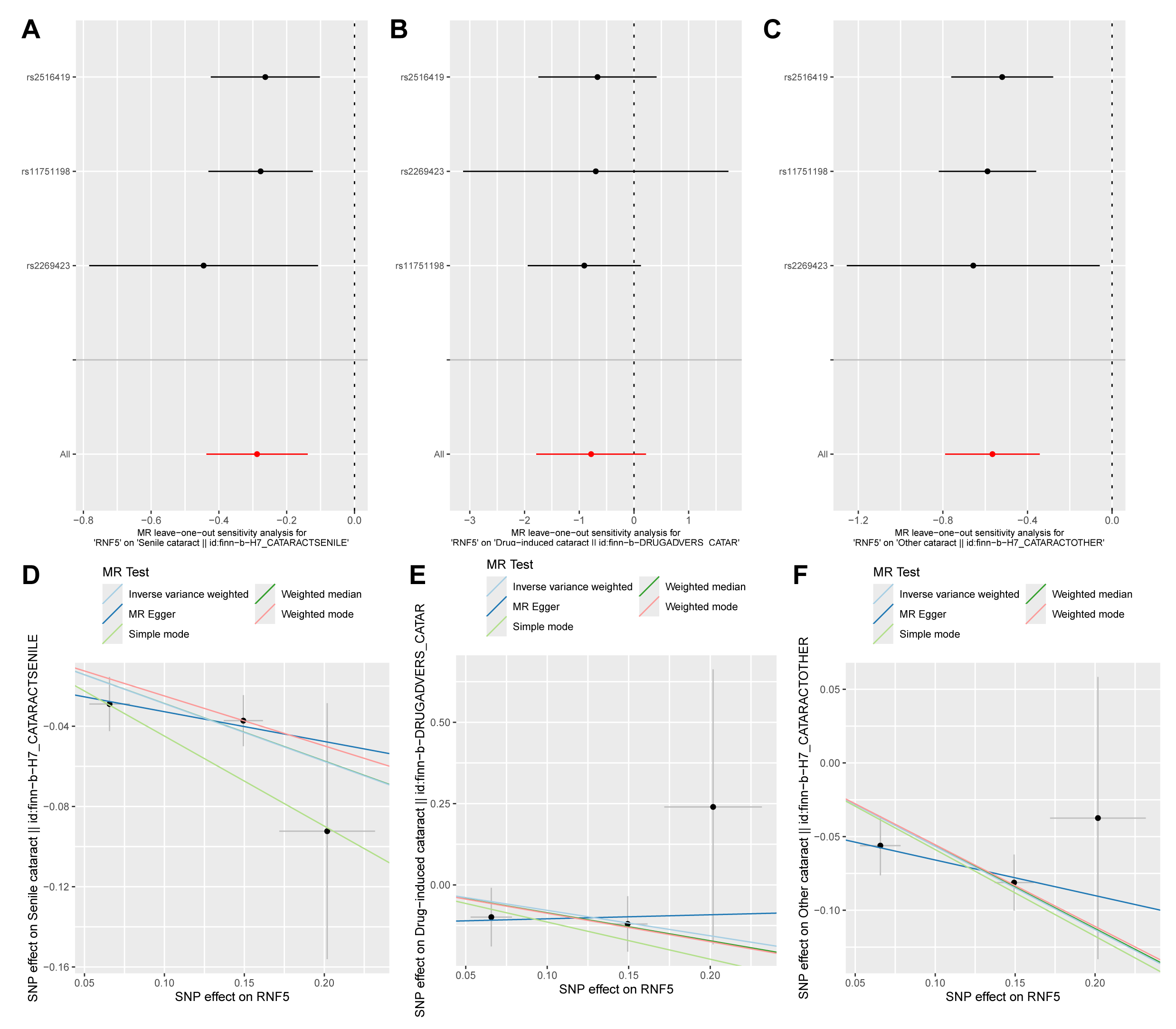


**Figure S6. The causal analysis between RNF5 and cataracts.** (A) Leave-one-out plots for the causal effect of RNF5 on other senile cataract. (B) Leave-one-out plots for the causal effect of RNF5 on drug-induced cataract. (C) Leave-one-out plots for the causal effect of RNF5 on other cataract. (D) Scatter plots for the causal effect of RNF5 on other senile cataract. (E) Scatter plots for the causal effect of RNF5 on drug-induced cataract. (F) Scatter plots for the causal effect of RNF5 on other cataract.


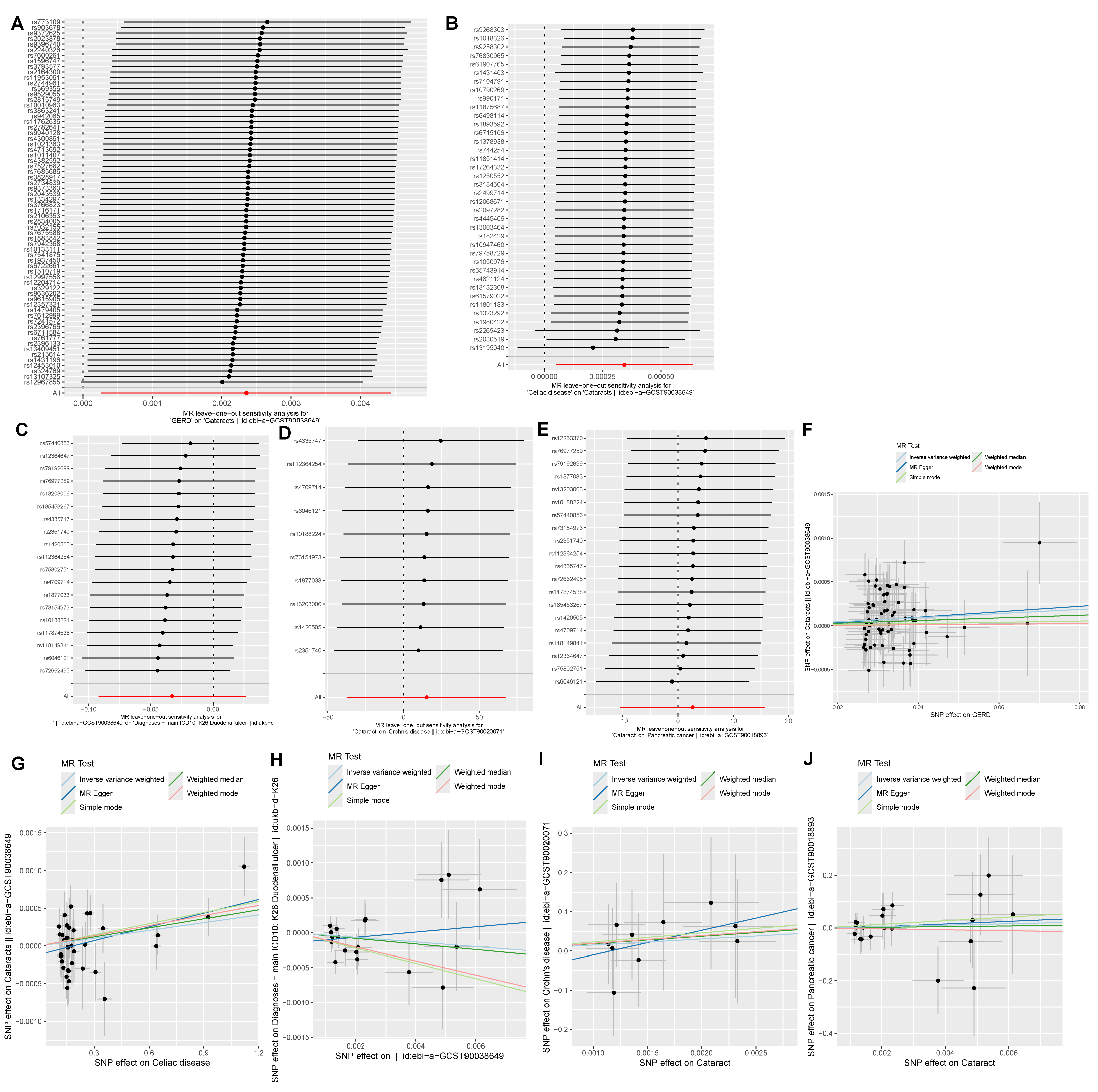


**Figure S7. The causal analysis of cataracts (ebi-a-GCST90038649) and gastrointestinal diseases.** (A) Leave-one-out plots for the causal effect of gastroesophageal reflux disease (GERD) on cataracts. (B) Leave-one-out plots for the causal effect of celiac disease on cataracts. (C) Leave-one-out plots for the causal effect of cataracts on duodenal ulcers. (D) Leave-one-out plots for the causal effect of cataracts on Crohn's disease. (E) Leave-one-out plots for the causal effect of cataracts on pancreatic cancer. (F) Scatter plots for the causal effect of GERD on cataracts. (G) Scatter plots for the causal effect of celiac disease on cataracts. (H) Scatter plots for the causal effect of cataracts on duodenal ulcers. (I) Scatter plots for the causal effect of cataracts on Crohn's disease. (J) Scatter plots for the causal effect of cataracts on pancreatic cancer.


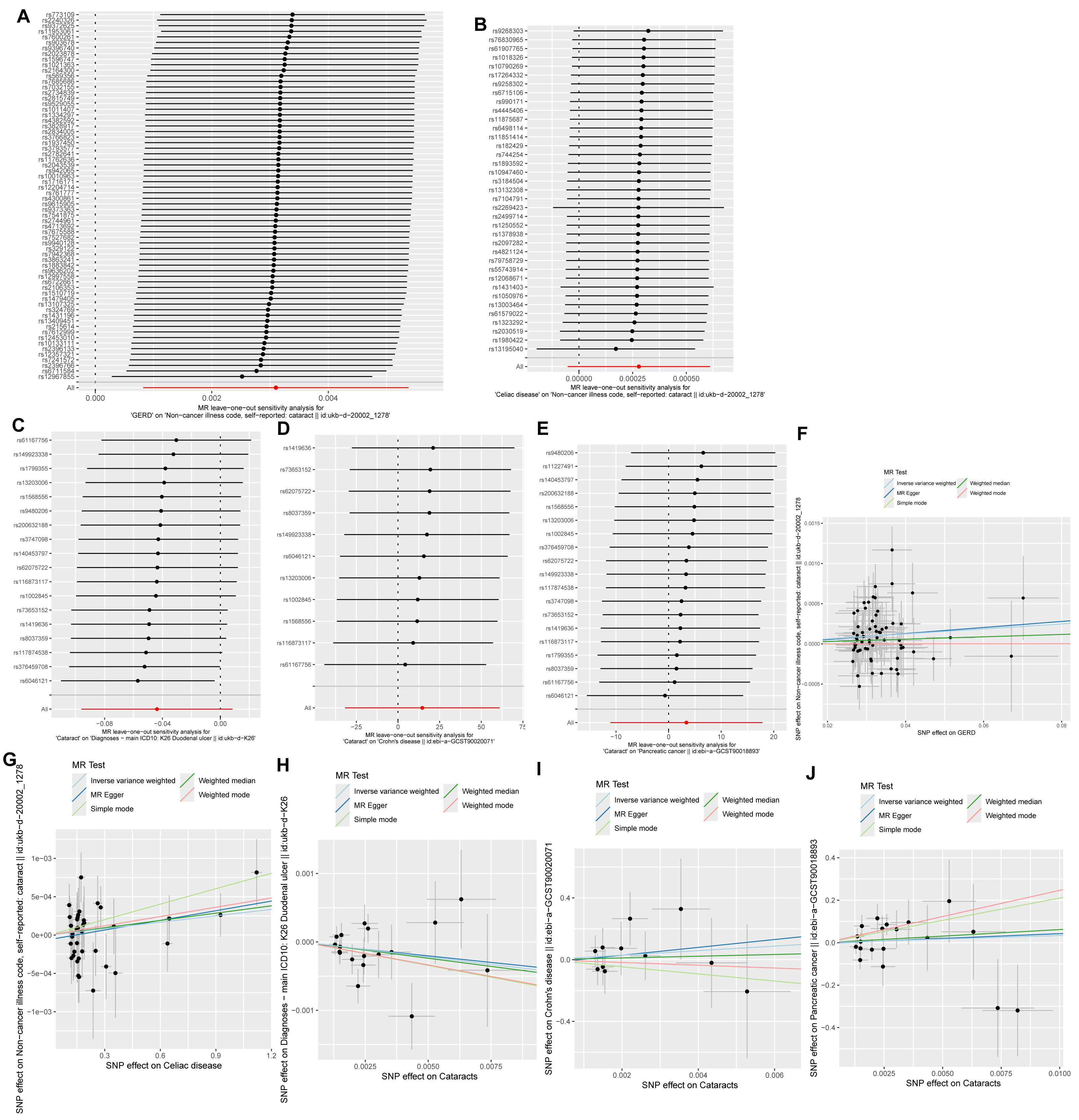


**Figure S8. The causal analysis of cataracts (ukb-d-20002_1278) and gastrointestinal diseases.** (A) Leave-one-out plots for the causal effect of gastroesophageal reflux disease (GERD) on cataracts. (B) Leave-one-out plots for the causal effect of celiac disease on cataracts. (C) Leave-one-out plots for the causal effect of cataracts on duodenal ulcers. (D) Leave-one-out plots for the causal effect of cataracts on Crohn's disease. (E) Leave-one-out plots for the causal effect of cataracts on pancreatic cancer. (F) Scatter plots for the causal effect of GERD on cataracts. (G) Scatter plots for the causal effect of celiac disease on cataracts. (H) Scatter plots for the causal effect of cataracts on duodenal ulcers. (I) Scatter plots for the causal effect of cataracts on Crohn's disease. (J) Scatter plots for the causal effect of cataracts on pancreatic cancer.
