# Supplementary material for: Causal Relationship Between Cataracts and Gastrointestinal Diseases: A Two-Sample Mendelian Randomization Study
Source: Transl Vis Sci Technol. 2025 Aug 20;14(8):27. doi: 10.1167/tvst.14.8.27 (PMC12372943; doi:10.1167/tvst.14.8.27)
Supplement: Supplement 2 [file tvst-14-8-27_s002.docx]

**Table S1 Detailed information on datasets used in this study.**

| **Exposure or outcome** | **GWAS ID** | **Sample** | **Case** | **Control** | **SNP** | **Population** | **Year** | ***P* value** |
| --- | --- | --- | --- | --- | --- | --- | --- | --- |
| Cataracts | ebi-a-GCST90018814 | 491877 | 39519 | 452358 | 24163031 | European | 2021 | 5E-08 |
| Gastroesophageal reflux disease | ebi-a-GCST90000514 | 602604 | 129080 | 473524 | 2320781 | European | 2021 | 5E-08 |
| Esophageal Cancer | ebi-a-GCST90018841 | 476306 | 998 | 475308 | 24194380 | European | 2021 | 5E-06 |
| Duodenal ulcer | ukb-d-K26 | 361194 | 1291 | 359903 | 9827456 | European | 2018 | 5E-06 |
| Gastric ulcer | ebi-a-GCST90018851 | 474278 | 6293 | 467985 | 24178780 | European | 2021 | 5E-06 |
| Acute gastritis | finn-b-K11_ACUTGASTR | 190979 | 1284 | 189695 | 16380389 | European | 2021 | 5E-06 |
| Chronic gastritis | ebi-a-GCST90018825 | 445096 | 3645 | 441451 | 24189505 | European | 2021 | 5E-06 |
| Gastric cancer | ebi-a-GCST90018849 | 476116 | 1029 | 475087 | 24188662 | European | 2021 | 5E-08 |
| Irritable bowel syndrome | ebi-a-GCST90016564 | 486601 | 53400 | 433201 | 9739966 | European | 2021 | 5E-08 |
| Celiac disease | ebi-a-GCST005523 | 23649 | 11812 | 229 | 97422 | European | 2011 | 5E-08 |
| Crohn’s disease | ebi-a-GCST90020071 | 1219 | 284 | 935 | 5394739 | European | 2022 | 5E-06 |
| Ulcerative Colitis | ebi-a-GCST90018933 | 417932 | 5371 | 412561 | 24187301 | European | 2021 | 5E-08 |
| Colorectal Cancer | ebi-a-GCST90018808 | 470002 | 6581 | 463421 | 24182361 | European | 2021 | 5E-08 |
| Diverticular disease | ukb-a-102 | 337159 | 3840 | 333319 | 10894596 | European | 2017 | 5E-06 |
| Acute pancreatitis | ebi-a-GCST90018789 | 479902 | 3798 | 476,10 | 24190697 | European | 2021 | 5E-06 |
| Chronic Pancreatitis | ebi-a-GCST90018821 | 477528 | 1424 | 476104 | 24195431 | European | 2021 | 5E-06 |
| Pancreatic Cancer | ebi-a-GCST90018893 | 476245 | 1196 | 475049 | 24195229 | European | 2021 | 5E-06 |
| Cholelithiasis | ebi-a-GCST90018819 | 487553 | 26122 | 461431 | 24173391 | European | 2021 | 5E-08 |
| Cholecystitis | ebi-a-GCST90018818 | 471251 | 9820 | 461431 | 24183039 | European | 2021 | 5E-08 |
| Nonalcoholic fatty liver disease | ebi-a-GCST90091033 | 778614 | 8434 | 770180 | 6784388 | European | 2021 | 5E-06 |
| Alcoholic liver disease | finn-b-ALCOLIVER | 218792 | 1416 | 217376 | 16380466 | European | 2021 | 5E-06 |
| Cirrhosis | ebi-a-GCST90018826 | 347406 | 122 | 347284 | 19079888 | European | 2021 | 5E-06 |
| Hepatic cancer | ebi-a-GCST90018858 | 475638 | 379 | 475259 | 24194938 | European | 2021 | 5E-08 |
| Acute appendicitis | finn-b-K11_APPENDACUT | 217513 | 15627 | 201886 | 16380463 | European | 2021 | 5E-08 |
| Senile cataract | finn-b-H7_CATARACTSENILE | 216362 | 26758 | 189604 | 16380461 | European | 2021 | 5E-08 |
| Drug-induced cataract | finn-b-DRUGADVERS_CATAR | 218792 | 330 | 218462 | 16380466 | European | 2021 | 5E-06 |
| Other cataract | finn-b-H7_CATARACTOTHER | 197477 | 7873 | 189604 | 16380403 | European | 2021 | 5E-06 |
| Cataracts | ebi-a-GCST90038649 | 484598 | 7038 | 477560 | 9587836 | European | 2021 | 5E-08 |
| Cataracts | ukb-d-20002_1278 | 361141 | 5045 | 356096 | 12055990 | European | 2018 | 5E-06 |
| 1400 plasma metabolites | GCST90199621-GCST90201020 |  |  |  |  | European | 2023 | 1E-05 |
| ieu-a-299 | HDL cholesterol | 187167 |  |  | 2447442 |  | 2013 | 5E-08 |
| ieu-a-300 | LDL cholesterol | 173082 |  |  | 2437752 |  | 2013 | 5E-08 |
| ieu-a-302 | Triglycerides | 177861 |  |  | 2439433 |  | 2013 | 5E-08 |
| ieu-a-301 | Total cholesterol | 187365 |  |  | 2446982 |  | 2013 | 5E-08 |
| RNF5 | eqtl-a-ENSG00000204308 | 8269 |  |  |  | European | 2018 | 5E-06 |

**Table S2. Definitions of cataract and 23 gastrointestinal diseases in the IEU GWAS database.**

| **Trait** | **GWAS** | **Reference** | **Case Definition** | **Control Definition** |
| --- | --- | --- | --- | --- |
| Cataracts | ebi-a-GCST90018814 | 34594039 | Clinical diagnosis identified through EMR, PMH, and phecode-mapped disease codes | Individuals without specific diagnoses or related phecodes |
| Esophageal Cancer | ebi-a-GCST90018841 |  |  |  |
| Gastric ulcer | ebi-a-GCST90018851 |  |  |  |
| Chronic gastritis | ebi-a-GCST90018825 |  |  |  |
| Gastric cancer | ebi-a-GCST90018849 |  |  |  |
| Ulcerative Colitis | ebi-a-GCST90018933 |  |  |  |
| Colorectal Cancer | ebi-a-GCST90018808 |  |  |  |
| Acute pancreatitis | ebi-a-GCST90018789 |  |  |  |
| Chronic Pancreatitis | ebi-a-GCST90018821 |  |  |  |
| Pancreatic Cancer | ebi-a-GCST90018893 |  |  |  |
| Cholelithiasis | ebi-a-GCST90018819 |  |  |  |
| Cholecystitis | ebi-a-GCST90018818 |  |  |  |
| Cirrhosis | ebi-a-GCST90018826 |  |  |  |
| Hepatic cancer | ebi-a-GCST90018858 |  |  |  |
| Gastroesophageal reflux disease | ebi-a-GCST90000514 | 34187846 | Self-report (Field ID: 20002), ICD-10/ICD-9 (Fields: 41202, 41204, 41203, 41205), operative records, medication data | No upper GI diagnosis or related medication |
| Celiac Disease | ebi-a-GCST005523 | 22057235 | Biopsy-confirmed Marsh II/III, with serological/HLA-DQ support | General population with no diagnosis or serological evidence |
| Crohn’s Disease | ebi-a-GCST90020071 | 35232999 | Confirmed diagnosis by location and behavior (inflammatory/stricturing/penetrating) | Non-IBD samples, passed strict QC filters (no kinship, HWE, PCA outliers) |
| Irritable bowel syndrome | ebi-a-GCST90016564 | 34741163 | Rome III criteria (DHQ), prompted/unprompted self-report, ICD-10 K58 (41202/41204) | DHQ controls (low symptom), non-respondent controls excluding GI diseases |
| Nonalcoholic fatty liver disease | ebi-a-GCST90091033 | 34841290 | ICD-10 codes: K76.0, K75.8, K76.9 etc. | No liver disease diagnosis; excludes alcohol-related liver disease, hepatitis, rare liver disorders |
| Alcoholic Liver Disease | finn-b-ALCOLIVER | Risteys: ALCOLIVER | ICD-10: K70, ICD-9: 571[0–3], ICD-8: 5710 | Individuals without matching diagnosis in EHR |
| Acute Gastritis | finn-b-K11_ACUTGASTR | Risteys: K11_ACUTGASTR | ICD-10: K29.0, K29.1; ICD-9: 5350; ICD-8: 3500[0-2] | Individuals without diagnosis codes in registry |
| Acute Appendicitis | finn-b-K11_APPENDACUT | Risteys: K11_APPENDACUT | ICD-10: K35; ICD-9/8: 540 | Individuals without diagnosis codes in registry |
| Duodenal ulcer | ukb-d-K26 | UK Biobank | ICD-10 K26-based hospital record | No diagnosis related to duodenal ulcer |
| Diverticular disease | ukb-a-102 | UK Biobank | ICD codes self-reported and/or PheCODE-based diagnosis | No history of diverticulosis/diverticulitis |

Definitions for UKB-derived traits (ukb-d/ukb-a) are based on general principles used in IEU OpenGWAS, as specific source documentation was not available.

EMR, electronic medical records; PHM, past medical history.

**Table S3 Mendelian randomization analysis of gastrointestinal diseases with cataracts.**

| **Exposure: 23 gastrointestinal diseases; Outcome: cataracts** | | | | | | | |
| --- | --- | --- | --- | --- | --- | --- | --- |
| **Gastroesophageal reflux disease** | **SNPs** | **Beta** | **SE** | ***p* value** | **or** | **or_lci95** | **or_uci95** |
| MR Egger | 66 | -0.122242102 | 0.180529389 | 0.500762982 | 0.884934098 | 0.621213967 | 1.260609709 |
| Weighted median | 66 | 0.078714712 | 0.041591431 | 0.058415018 | 1.081895627 | 0.997199437 | 1.173785407 |
| Inverse variance weighted | 66 | 0.123261022 | 0.030671345 | **5.85E-05** | 1.131179645 | 1.065181477 | 1.20126703 |
| Simple mode | 66 | 0.039148851 | 0.104437977 | 0.708990359 | 1.039925267 | 0.847427843 | 1.276149431 |
| Weighted mode | 66 | 0.046145451 | 0.099010623 | 0.642726607 | 1.04722672 | 0.862504106 | 1.271511401 |
| **Esophageal Cancer** | **SNPs** | **Beta** | **SE** | ***p* value** | **or** | **or_lci95** | **or_uci95** |
| MR Egger | 22 | -0.011589253 | 0.018126872 | 0.529856592 | 0.988477644 | 0.953974897 | 1.024228264 |
| Weighted median | 22 | 0.011986565 | 0.011648328 | 0.303462084 | 1.012058692 | 0.989214429 | 1.035430506 |
| Inverse variance weighted | 22 | 0.004435954 | 0.009025194 | 0.623067262 | 1.004445808 | 0.986834014 | 1.022371915 |
| Simple mode | 22 | 0.01120963 | 0.019653906 | 0.574491884 | 1.011272694 | 0.973057576 | 1.050988643 |
| Weighted mode | 22 | 0.011794852 | 0.014977036 | 0.439771002 | 1.011864685 | 0.982593142 | 1.042008231 |
| **Duodenal ulcer** | **SNPs** | **Beta** | **SE** | ***p* value** | **or** | **or_lci95** | **or_uci95** |
| MR Egger | 15 | -2.155274561 | 6.567780934 | 0.748017123 | 0.115871372 | 2.97E-07 | 45142.34818 |
| Weighted median | 15 | -0.297424976 | 3.849785828 | 0.938418669 | 0.742728303 | 0.000392488 | 1405.509637 |
| Inverse variance weighted | 15 | -2.085219427 | 3.07592076 | 0.497824227 | 0.124279847 | 0.000299312 | 51.60328138 |
| Simple mode | 15 | -2.651585261 | 6.736806373 | 0.699807911 | 0.070539301 | 1.30E-07 | 38275.11521 |
| Weighted mode | 15 | -0.224880649 | 4.678086676 | 0.962338639 | 0.798611528 | 8.32E-05 | 7663.079741 |
| **Gastric ulcer** | **SNPs** | **Beta** | **SE** | ***p* value** | **or** | **or_lci95** | **or_uci95** |
| MR Egger | 26 | 0.04017749 | 0.046443738 | 0.395560555 | 1.040995524 | 0.950419097 | 1.140204025 |
| Weighted median | 26 | -0.023891373 | 0.030505238 | 0.43351602 | 0.976391767 | 0.919724014 | 1.036551039 |
| Inverse variance weighted | 26 | -0.01497114 | 0.021452589 | 0.485257886 | 0.985140371 | 0.944576864 | 1.02744582 |
| Simple mode | 26 | -0.018656706 | 0.055461169 | 0.739382432 | 0.981516253 | 0.880416139 | 1.094225914 |
| Weighted mode | 26 | -0.038791043 | 0.049258871 | 0.438395835 | 0.961951694 | 0.87342027 | 1.059456821 |
| **Acute gastritis** | **SNPs** | **Beta** | **SE** | ***p* value** | **or** | **or_lci95** | **or_uci95** |
| MR Egger | 5 | -0.017735813 | 0.047837818 | 0.735456541 | 0.982420541 | 0.894493237 | 1.078990963 |
| Weighted median | 5 | -0.021525762 | 0.014499619 | 0.137656623 | 0.978704264 | 0.951281729 | 1.006917306 |
| Inverse variance weighted | 5 | -0.021584761 | 0.012419708 | 0.082220635 | 0.978646523 | 0.955111311 | 1.002761674 |
| Simple mode | 5 | -0.014997116 | 0.021641475 | 0.52645473 | 0.985114781 | 0.944202704 | 1.027799567 |
| Weighted mode | 5 | -0.011116335 | 0.020919404 | 0.623288198 | 0.988945223 | 0.949216505 | 1.030336756 |
| **Chronic gastritis** | **SNPs** | **Beta** | **SE** | ***p* value** | **or** | **or_lci95** | **or_uci95** |
| MR Egger | 19 | 0.015042151 | 0.028575442 | 0.60541001 | 1.015155854 | 0.959862038 | 1.073634926 |
| Weighted median | 19 | 0.016714488 | 0.014683809 | 0.254997843 | 1.016854956 | 0.988006722 | 1.046545514 |
| Inverse variance weighted | 19 | 0.005265894 | 0.014340765 | 0.713471642 | 1.005279783 | 0.977416899 | 1.033936945 |
| Simple mode | 19 | -0.008080371 | 0.022376981 | 0.722226708 | 0.991952188 | 0.949386537 | 1.036426265 |
| Weighted mode | 19 | 0.016775219 | 0.017751717 | 0.357176589 | 1.016916713 | 0.98214321 | 1.052921398 |
| **Gastric cancer** | **SNPs** | **Beta** | **SE** | ***p* value** | **or** | **or_lci95** | **or_uci95** |
| MR Egger | 7 | 0.241474274 | 0.109264113 | 0.078103958 | 1.273124702 | 1.027693131 | 1.577169739 |
| Weighted median | 7 | 0.012732861 | 0.031759315 | 0.688480963 | 1.012814269 | 0.951690506 | 1.077863798 |
| Inverse variance weighted | 7 | -0.026281399 | 0.037962443 | 0.488748944 | 0.974060951 | 0.904215297 | 1.049301798 |
| Simple mode | 7 | 0.006391963 | 0.04620435 | 0.894496996 | 1.006412435 | 0.919276286 | 1.101808025 |
| Weighted mode | 7 | 0.011202809 | 0.033789412 | 0.751504991 | 1.011265795 | 0.946462017 | 1.080506655 |
| **Irritable bowel syndrome** | **SNPs** | **Beta** | **SE** | ***p* value** | **or** | **or_lci95** | **or_uci95** |
| MR Egger | 4 | -1.719204146 | 2.411887824 | 0.549910603 | 0.179208715 | 0.001586057 | 20.24880923 |
| Weighted median | 4 | 0.040860154 | 0.1110088 | 0.712813346 | 1.041706417 | 0.838016832 | 1.294905088 |
| Inverse variance weighted | 4 | -0.091190809 | 0.163710329 | 0.577510085 | 0.912843515 | 0.662282515 | 1.258199128 |
| Simple mode | 4 | 0.123863479 | 0.258419338 | 0.664488024 | 1.131861338 | 0.682059471 | 1.87829675 |
| Weighted mode | 4 | 0.131853045 | 0.176368425 | 0.508966919 | 1.140940639 | 0.807486398 | 1.612095939 |
| **Celiac disease** | **SNPs** | **Beta** | **SE** | ***p* value** | **or** | **or_lci95** | **or_uci95** |
| MR Egger | 38 | 0.020586718 | 0.006082066 | **0.001732825** | 1.020800086 | 1.008703526 | 1.03304171 |
| Weighted median | 38 | 0.017121134 | 0.006406293 | **0.007527733** | 1.01726854 | 1.004575234 | 1.030122233 |
| Inverse variance weighted | 38 | 0.012130915 | 0.003981832 | **0.002314712** | 1.012204793 | 1.004335898 | 1.020135341 |
| Simple mode | 38 | 0.00684392 | 0.01264101 | 0.591473066 | 1.006867393 | 0.982227369 | 1.032125533 |
| Weighted mode | 38 | 0.011705294 | 0.005481687 | **0.039418617** | 1.011774069 | 1.000961649 | 1.022703284 |
| **Crohn's disease** | **SNPs** | **Beta** | **SE** | ***p* value** | **or** | **or_lci95** | **or_uci95** |
| MR Egger | 12 | -0.001262322 | 0.024608349 | 0.960099467 | 0.998738475 | 0.951710216 | 1.04809061 |
| Weighted median | 12 | 0.000404732 | 0.005350742 | 0.939705214 | 1.000404814 | 0.989967939 | 1.010951721 |
| Inverse variance weighted | 12 | -0.001712569 | 0.004114975 | 0.677278552 | 0.998288897 | 0.990269728 | 1.006373004 |
| Simple mode | 12 | 0.003208511 | 0.008615142 | 0.716650778 | 1.003213664 | 0.986415941 | 1.020297437 |
| Weighted mode | 12 | 0.003208511 | 0.008114208 | 0.700094389 | 1.003213664 | 0.98738491 | 1.019296169 |
| **Ulcerative Colitis** | **SNPs** | **Beta** | **SE** | ***p* value** | **or** | **or_lci95** | **or_uci95** |
| MR Egger | 16 | -0.025374005 | 0.045599066 | 0.586682302 | 0.974945209 | 0.891590641 | 1.066092573 |
| Weighted median | 16 | 0.014670627 | 0.018986443 | 0.439706088 | 1.014778769 | 0.97770939 | 1.053253617 |
| Inverse variance weighted | 16 | -0.000466015 | 0.018970853 | 0.9804021 | 0.999534093 | 0.96305102 | 1.037399248 |
| Simple mode | 16 | 0.047924581 | 0.034767357 | 0.188281942 | 1.049091531 | 0.979983604 | 1.123072912 |
| Weighted mode | 16 | 0.029182048 | 0.027545689 | 0.306177366 | 1.029612016 | 0.975497674 | 1.086728274 |
| **Colorectal Cancer** | **SNPs** | **Beta** | **SE** | ***p* value** | **or** | **or_lci95** | **or_uci95** |
| MR Egger | 26 | 0.034217182 | 0.152694936 | 0.82458512 | 1.034809325 | 0.767156166 | 1.39584401 |
| Weighted median | 26 | -0.00189837 | 0.022609269 | 0.933084861 | 0.998103431 | 0.954839001 | 1.043328202 |
| Inverse variance weighted | 26 | -0.010453669 | 0.032152553 | 0.745084817 | 0.989600781 | 0.929161537 | 1.053971421 |
| Simple mode | 26 | 0.024790426 | 0.03697298 | 0.508685642 | 1.025100264 | 0.953442066 | 1.102144104 |
| Weighted mode | 26 | 0.02002355 | 0.032343279 | 0.541455071 | 1.020225366 | 0.957557722 | 1.086994314 |
| **Diverticular disease** | **SNPs** | **Beta** | **SE** | ***p* value** | **or** | **or_lci95** | **or_uci95** |
| MR Egger | 19 | -2.568628899 | 3.147027863 | 0.425671022 | 0.076640555 | 0.000160566 | 36.5816121 |
| Weighted median | 19 | -1.49774679 | 1.557080169 | 0.336102621 | 0.223633486 | 0.010571179 | 4.730970407 |
| Inverse variance weighted | 19 | -0.104552744 | 1.094668298 | 0.923909041 | 0.900727289 | 0.10538841 | 7.698281484 |
| Simple mode | 19 | -2.052094814 | 2.25999088 | 0.375869741 | 0.12846551 | 0.001531241 | 10.77778759 |
| Weighted mode | 19 | -2.282671309 | 1.860649672 | 0.235698205 | 0.102011339 | 0.002659693 | 3.912599743 |
| **Acute pancreatitis** | **SNPs** | **Beta** | **SE** | ***p* value** | **or** | **or_lci95** | **or_uci95** |
| MR Egger | 15 | -0.021152773 | 0.041369217 | 0.617702694 | 0.979069377 | 0.902816066 | 1.061763167 |
| Weighted median | 15 | 0.018240856 | 0.02286728 | 0.425054071 | 1.018408237 | 0.9737711 | 1.065091516 |
| Inverse variance weighted | 15 | 0.022721822 | 0.018420479 | 0.217386016 | 1.022981929 | 0.986706828 | 1.060590641 |
| Simple mode | 15 | 0.02036936 | 0.038268043 | 0.602876999 | 1.020578231 | 0.946829728 | 1.100071 |
| Weighted mode | 15 | 0.012118938 | 0.030920957 | 0.701009164 | 1.01219267 | 0.952670546 | 1.075433691 |
| **Chronic Pancreatitis** | **SNPs** | **Beta** | **SE** | ***p* value** | **or** | **or_lci95** | **or_uci95** |
| MR Egger | 15 | 0.006368355 | 0.02143813 | 0.771116018 | 1.006388676 | 0.964977611 | 1.049576857 |
| Weighted median | 15 | -0.005560671 | 0.01407135 | 0.69271243 | 0.994454761 | 0.967402614 | 1.022263386 |
| Inverse variance weighted | 15 | -0.000559089 | 0.011903559 | 0.962538537 | 0.999441067 | 0.976393044 | 1.023033145 |
| Simple mode | 15 | -0.022307404 | 0.029136746 | 0.456622231 | 0.977939567 | 0.923656134 | 1.035413246 |
| Weighted mode | 15 | -0.016493786 | 0.02498089 | 0.519805593 | 0.983641492 | 0.936639949 | 1.03300162 |
| **Pancreatic Cancer** | **SNPs** | **Beta** | **SE** | ***p* value** | **or** | **or_lci95** | **or_uci95** |
| MR Egger | 15 | 0.021710219 | 0.027239064 | 0.439751187 | 1.0219476 | 0.968818156 | 1.077990633 |
| Weighted median | 15 | -0.008350653 | 0.01440083 | 0.562000254 | 0.991684117 | 0.96408455 | 1.020073797 |
| Inverse variance weighted | 15 | -0.002624627 | 0.012696576 | 0.836229071 | 0.997378814 | 0.972865035 | 1.022510279 |
| Simple mode | 15 | -0.029607779 | 0.029952113 | 0.339683398 | 0.970826238 | 0.915473445 | 1.029525857 |
| Weighted mode | 15 | -0.027755153 | 0.026591824 | 0.314290764 | 0.972626482 | 0.923231625 | 1.024664069 |
| **Cholelithiasis** | **SNPs** | **Beta** | **SE** | ***p* value** | **or** | **or_lci95** | **or_uci95** |
| MR Egger | 42 | 0.003977519 | 0.021691982 | 0.855439087 | 1.00398544 | 0.962194404 | 1.047591587 |
| Weighted median | 42 | -5.67E-05 | 0.017840632 | 0.997463198 | 0.999943279 | 0.965581893 | 1.035527456 |
| Inverse variance weighted | 42 | 0.002645359 | 0.013645706 | 0.846285357 | 1.002648861 | 0.976187868 | 1.029827118 |
| Simple mode | 42 | -0.025060781 | 0.032462178 | 0.444542026 | 0.975250633 | 0.915132285 | 1.039318373 |
| Weighted mode | 42 | -0.003239719 | 0.015622679 | 0.836745274 | 0.996765523 | 0.966706671 | 1.027759028 |
| **Cholecystitis** | **SNPs** | **Beta** | **SE** | ***p* value** | **or** | **or_lci95** | **or_uci95** |
| MR Egger | 15 | -0.005354852 | 0.025140739 | 0.834634586 | 0.994659459 | 0.946834749 | 1.0448998 |
| Weighted median | 15 | 0.004410067 | 0.020363057 | 0.828541965 | 1.004419805 | 0.965121262 | 1.045318536 |
| Inverse variance weighted | 15 | 0.014376851 | 0.015046501 | 0.339327606 | 1.014480695 | 0.984999357 | 1.044844418 |
| Simple mode | 15 | 0.051745598 | 0.037813915 | 0.19273876 | 1.053107795 | 0.977878669 | 1.134124369 |
| Weighted mode | 15 | 0.009329344 | 0.020405226 | 0.654538829 | 1.009372998 | 0.9698005 | 1.050560244 |
| **Nonalcoholic fatty liver disease** | **SNPs** | **Beta** | **SE** | **p value** | **or** | **or_lci95** | **or_uci95** |
| MR Egger | 9 | -0.011675639 | 0.046976192 | 0.810847929 | 0.988392257 | 0.901451555 | 1.083717974 |
| Weighted median | 9 | 0.011744526 | 0.021383794 | 0.582850689 | 1.011813763 | 0.970282793 | 1.055122382 |
| Inverse variance weighted | 9 | 0.034676199 | 0.024363191 | 0.154648378 | 1.035284429 | 0.98700946 | 1.085920542 |
| Simple mode | 9 | 0.001214785 | 0.045424278 | 0.979319727 | 1.001215523 | 0.91592866 | 1.094443889 |
| Weighted mode | 9 | 0.006938014 | 0.021225439 | 0.752152585 | 1.006962138 | 0.965930063 | 1.049737227 |
| **Alcoholic liver disease** | **SNPs** | **Beta** | **SE** | ***p* value** | **or** | **or_lci95** | **or_uci95** |
| MR Egger | 11 | -0.023102868 | 0.023990846 | 0.360712717 | 0.977161959 | 0.932277347 | 1.024207548 |
| Weighted median | 11 | 0.00370125 | 0.011700647 | 0.751753082 | 1.003708108 | 0.980951736 | 1.026992388 |
| Inverse variance weighted | 11 | -0.002428468 | 0.011383484 | 0.831067546 | 0.997574478 | 0.975563431 | 1.020082147 |
| Simple mode | 11 | -0.000865732 | 0.017235027 | 0.960927485 | 0.999134642 | 0.96594693 | 1.033462609 |
| Weighted mode | 11 | 0.002119525 | 0.013605155 | 0.879299783 | 1.002121773 | 0.975752238 | 1.029203939 |
| **Cirrhosis** | **SNPs** | **Beta** | **SE** | ***p* value** | **or** | **or_lci95** | **or_uci95** |
| MR Egger | 18 | -0.00750798 | 0.007900708 | 0.356098757 | 0.992520134 | 0.977268966 | 1.008009312 |
| Weighted median | 18 | -0.005059154 | 0.006932056 | 0.465500144 | 0.994953622 | 0.981526777 | 1.00856414 |
| Inverse variance weighted | 18 | -0.000809495 | 0.00619323 | 0.896007485 | 0.999190832 | 0.987135242 | 1.011393654 |
| Simple mode | 18 | 0.004317094 | 0.009594869 | 0.658439819 | 1.004326426 | 0.985615609 | 1.023392446 |
| Weighted mode | 18 | -0.003879738 | 0.006822349 | 0.577009047 | 0.996127779 | 0.982896415 | 1.009537258 |
| **Hepatic cancer** | **SNPs** | **Beta** | **SE** | ***p* value** | **or** | **or_lci95** | **or_uci95** |
| MR Egger | 5 | 0.019483544 | 0.0266472 | 0.517610087 | 1.019674587 | 0.967785345 | 1.074345948 |
| Weighted median | 5 | 0.008494043 | 0.013740032 | 0.536445654 | 1.00853022 | 0.981732491 | 1.03605943 |
| Inverse variance weighted | 5 | -0.007558141 | 0.018562029 | 0.68387365 | 0.99247035 | 0.957011646 | 1.029242851 |
| Simple mode | 5 | 0.003786934 | 0.018490593 | 0.847725172 | 1.003794113 | 0.968066372 | 1.040840433 |
| Weighted mode | 5 | 0.007204784 | 0.013797285 | 0.62912303 | 1.007230801 | 0.980357583 | 1.034840659 |
| **Acute appendicitis** | **SNPs** | **Beta** | **SE** | ***p* value** | **or** | **or_lci95** | **or_uci95** |
| MR Egger | 6 | 0.005296935 | 0.117902409 | 0.966319332 | 1.005310989 | 0.797884215 | 1.266662712 |
| Weighted median | 6 | -0.060269061 | 0.036879533 | 0.102213859 | 0.941511175 | 0.875856559 | 1.012087292 |
| Inverse variance weighted | 6 | -0.055955977 | 0.032311906 | 0.083318999 | 0.945580763 | 0.88755276 | 1.007402623 |
| Simple mode | 6 | -0.103361722 | 0.063897855 | 0.166670666 | 0.901800714 | 0.795645522 | 1.022119154 |
| Weighted mode | 6 | -0.054191 | 0.045412408 | 0.286277661 | 0.947251164 | 0.866581326 | 1.035430538 |

Beta, regression coefficient. SE, Standard Error. or, odds ratio. or_lci95, lower confidence interval of 95% for the odds ratio. or_uci95, upper confidence agreement of 95% for the odds ratio.

**Table S4 Horizontal pleiotropy test**

| **Outcome** | **Exposure** | **Egger_intercept** | **SE** | ***P* value** |
| --- | --- | --- | --- | --- |
| Cataracts | Gastroesophageal reflux disease | 0.008098 | 0.00587 | 0.172484 |
| Cataracts | Esophageal Cancer | 0.005488 | 0.005385 | 0.320333 |
| Cataracts | Duodenal ulcer | 0.000115 | 0.009395 | 0.990447 |
| Cataracts | Gastric ulcer | -0.00549 | 0.004116 | 0.194931 |
| Cataracts | Acute gastritis | -0.0012 | 0.014402 | 0.938849 |
| Cataracts | Chronic gastritis | -0.00236 | 0.005912 | 0.694975 |
| Cataracts | Gastric cancer | -0.03719 | 0.014688 | 0.052389 |
| Cataracts | Irritable bowel syndrome | 0.066889 | 0.098816 | 0.568265 |
| Cataracts | Celiac disease | -0.0035 | 0.001902 | 0.074139 |
| Cataracts | Crohn's disease | -0.00025 | 0.01344 | 0.985559 |
| Cataracts | Ulcerative Colitis | 0.004354 | 0.007215 | 0.555832 |
| Cataracts | Colorectal Cancer | -0.00455 | 0.015188 | 0.76712 |
| Cataracts | Diverticular disease | 0.005713 | 0.00684 | 0.415224 |
| Cataracts | Acute pancreatitis | 0.008417 | 0.00713 | 0.25896 |
| Cataracts | Chronic Pancreatitis | -0.00274 | 0.006959 | 0.699813 |
| Cataracts | Pancreatic Cancer | -0.00749 | 0.00742 | 0.331113 |
| Cataracts | Cholelithiasis | -0.00018 | 0.002257 | 0.936911 |
| Cataracts | Cholecystitis | 0.003692 | 0.003766 | 0.344765 |
| Cataracts | Nonalcoholic fatty liver disease | 0.0074 | 0.006457 | 0.28941 |
| Cataracts | Alcoholic liver disease | 0.009502 | 0.0097 | 0.352897 |
| Cataracts | Cirrhosis | 0.003982 | 0.003012 | 0.204811 |
| Cataracts | Hepatic cancer | -0.01229 | 0.009306 | 0.278437 |
| Cataracts | Acute appendicitis | -0.00632 | 0.011626 | 0.615446 |

**Table S5 Heterogeneity test of the association between 23 gastrointestinal diseases with cataracts**

| **Outcome** | **Exposure** | **Method** | **Q** | **Q_df** | **Q_pval** |
| --- | --- | --- | --- | --- | --- |
| Cataracts | Gastroesophageal reflux disease | MR Egger | 79.12904 | 64 | 0.096415 |
| Cataracts | Gastroesophageal reflux disease | Inverse variance weighted | 81.48256 | 65 | 0.081328 |
| Cataracts | Esophageal Cancer | MR Egger | 30.26022 | 20 | 0.065745 |
| Cataracts | Esophageal Cancer | Inverse variance weighted | 31.83153 | 21 | 0.060889 |
| Cataracts | Duodenal ulcer | MR Egger | 19.79514 | 13 | 0.100437 |
| Cataracts | Duodenal ulcer | Inverse variance weighted | 19.79537 | 14 | 0.136726 |
| Cataracts | Gastric ulcer | MR Egger | 26.36342 | 24 | 0.334986 |
| Cataracts | Gastric ulcer | Inverse variance weighted | 28.31636 | 25 | 0.293497 |
| Cataracts | Acute gastritis | MR Egger | 0.94467 | 3 | 0.814637 |
| Cataracts | Acute gastritis | Inverse variance weighted | 0.951611 | 4 | 0.917042 |
| Cataracts | Chronic gastritis | MR Egger | 30.12778 | 17 | **0.025438** |
| Cataracts | Chronic gastritis | Inverse variance weighted | 30.40969 | 18 | **0.033649** |
| Cataracts | Gastric cancer | MR Egger | 6.917294 | 5 | 0.226864 |
| Cataracts | Gastric cancer | Inverse variance weighted | 15.78814 | 6 | **0.014937** |
| Cataracts | Irritable bowel syndrome | MR Egger | 8.862936 | 2 | **0.011897** |
| Cataracts | Irritable bowel syndrome | Inverse variance weighted | 10.89341 | 3 | **0.012316** |
| Cataracts | Celiac disease | MR Egger | 32.61439 | 36 | 0.63041 |
| Cataracts | Celiac disease | Inverse variance weighted | 35.99719 | 37 | 0.515903 |
| Cataracts | Crohn's disease | MR Egger | 6.676693 | 10 | 0.755574 |
| Cataracts | Crohn's disease | Inverse variance weighted | 6.677038 | 11 | 0.824602 |
| Cataracts | Ulcerative Colitis | MR Egger | 37.22333 | 14 | **0.000682** |
| Cataracts | Ulcerative Colitis | Inverse variance weighted | 38.19169 | 15 | **0.000845** |
| Cataracts | Colorectal Cancer | MR Egger | 126.9515 | 24 | **5.51E-16** |
| Cataracts | Colorectal Cancer | Inverse variance weighted | 127.426 | 25 | **1.06E-15** |
| Cataracts | Diverticular disease | MR Egger | 16.66616 | 17 | 0.477196 |
| Cataracts | Diverticular disease | Inverse variance weighted | 17.36362 | 18 | 0.498253 |
| Cataracts | Acute pancreatitis | MR Egger | 16.78423 | 13 | 0.209356 |
| Cataracts | Acute pancreatitis | Inverse variance weighted | 18.58341 | 14 | 0.181486 |
| Cataracts | Chronic Pancreatitis | MR Egger | 21.55879 | 13 | 0.062587 |
| Cataracts | Chronic Pancreatitis | Inverse variance weighted | 21.81651 | 14 | 0.082464 |
| Cataracts | Pancreatic Cancer | MR Egger | 22.90877 | 13 | **0.042784** |
| Cataracts | Pancreatic Cancer | Inverse variance weighted | 24.70495 | 14 | **0.037594** |
| Cataracts | Cholelithiasis | MR Egger | 59.81007 | 40 | **0.022737** |
| Cataracts | Cholelithiasis | Inverse variance weighted | 59.81955 | 41 | **0.028951** |
| Cataracts | Cholecystitis | MR Egger | 13.67077 | 13 | 0.397432 |
| Cataracts | Cholecystitis | Inverse variance weighted | 14.68163 | 14 | 0.400258 |
| Cataracts | Nonalcoholic fatty liver disease | MR Egger | 12.66602 | 7 | 0.080676 |
| Cataracts | Nonalcoholic fatty liver disease | Inverse variance weighted | 15.04288 | 8 | 0.058317 |
| Cataracts | Alcoholic liver disease | MR Egger | 17.73818 | 9 | **0.038336** |
| Cataracts | Alcoholic liver disease | Inverse variance weighted | 19.62936 | 10 | **0.03296** |
| Cataracts | Cirrhosis | MR Egger | 22.98988 | 16 | 0.114005 |
| Cataracts | Cirrhosis | Inverse variance weighted | 25.50045 | 17 | 0.084055 |
| Cataracts | Hepatic cancer | MR Egger | 6.839136 | 3 | 0.077206 |
| Cataracts | Hepatic cancer | Inverse variance weighted | 10.81296 | 4 | **0.028748** |
| Cataracts | Acute appendicitis | MR Egger | 5.620967 | 4 | 0.229299 |
| Cataracts | Acute appendicitis | Inverse variance weighted | 6.036579 | 5 | 0.302676 |

**Table S6 Correction for causal effects between cataracts and gastrointestinal diseases (after heterogeneity test)**

| **Outcome** | **Exposure** | **Random effect IVW** | | | | **MR-PRESSO tests/ *p* value** | |
| --- | --- | --- | --- | --- | --- | --- | --- |
|  |  | ***p* value** | **or** | **or_lci95** | **or_uci95** | **Raw** | **Outlier-corrected** |
| Cataracts | Chronic gastritis | 0.713471642 | 1.005279783 | 0.977416899 | 1.033936945 | 0.718 | NA |
| Cataracts | Gastric cancer | 0.488748944 | 0.974060951 | 0.904215297 | 1.049301798 | 0.515 | 0.879 |
| Cataracts | Irritable bowel syndrome | 0.577510085 | 0.912843515 | 0.662282515 | 1.258199128 | 0.616 | NA |
| Cataracts | Ulcerative Colitis | 0.9804021 | 0.999534093 | 0.96305102 | 1.037399248 | 0.981 | 0.171 |
| Cataracts | Colorectal Cancer | 0.745084817 | 0.989600781 | 0.929161537 | 1.053971421 | 0.748 | 0.210 |
| Cataracts | Pancreatic Cancer | 0.836229071 | 0.997378814 | 0.972865035 | 1.022510279 | 0.839 | NA |
| Cataracts | Cholelithiasis | 0.846285357 | 1.002648861 | 0.976187868 | 1.029827118 | 0.847 | NA |
| Cataracts | Alcoholic liver disease | 0.831067546 | 0.997574478 | 0.975563431 | 1.020082147 | 0.835 | 0.507 |
| Cataracts | Hepatic cancer | 0.68387365 | 0.99247035 | 0.957011646 | 1.029242851 | 0.705 | NA |
| Gastroesophageal reflux disease | Cataracts | 0.685207728 | 1.033479786 | 0.881353445 | 1.21186395 | 0.700 | 0.963 |
| Irritable bowel syndrome | Cataracts | 0.45025183 | 1.024542238 | 0.962041665 | 1.09110326 | 0.456 | NA |
| Ulcerative colitis | Cataracts | 0.404167387 | 0.872842269 | 0.634105168 | 1.201462573 | 0.412 | 0.634 |
| Cholelithiasis | Cataracts | 0.092172622 | 1.076590333 | 0.987979899 | 1.173148104 | 0.105 | 0.209 |
| Cholecystitis | Cataracts | 0.164716121 | 1.099948946 | 0.961630894 | 1.258162245 | 0.177 | 0.060 |
| Hepatic cancer | Cataracts | 0.106547972 | 1.390239108 | 0.931783684 | 2.074263383 | 0.120 | 0.065 |

or, odds ratio. or_lci95, lower confidence interval of 95% for the odds ratio. or_uci95, upper confidence agreement of 95% for the odds ratio. NA, No outliers detected.

**Table S7 Mendelian randomization analysis of cataracts with gastrointestinal diseases.**

| **Exposure: cataracts; Outcome: 23 gastrointestinal diseases** | | | | | | | |
| --- | --- | --- | --- | --- | --- | --- | --- |
| **Gastroesophageal reflux disease** | **SNPs** | **Beta** | **SE** | ***p* value** | **or** | **or_lci95** | **or_uci95** |
| MR Egger | 7 | 0.015080042 | 0.383716642 | 0.970172171 | 1.01519432 | 0.478545216 | 2.153651257 |
| Weighted median | 7 | 0.028556984 | 0.056064862 | 0.610502587 | 1.028968644 | 0.921889284 | 1.148485494 |
| Inverse variance weighted | 7 | 0.032931541 | 0.08123882 | 0.685207728 | 1.033479786 | 0.881353445 | 1.21186395 |
| Simple mode | 7 | -0.003540215 | 0.106178019 | 0.974483162 | 0.996466044 | 0.809248587 | 1.226995749 |
| Weighted mode | 7 | 0.047706102 | 0.072924466 | 0.537245074 | 1.048862352 | 0.909167575 | 1.210021412 |
| **Esophageal Cancer** | **SNPs** | **Beta** | **SE** | ***p* value** | **or** | **or_lci95** | **or_uci95** |
| MR Egger | 25 | -0.739022854 | 0.382800495 | 0.065955803 | 0.477580353 | 0.225527805 | 1.011329817 |
| Weighted median | 25 | -0.048454846 | 0.190329766 | 0.799044692 | 0.952700357 | 0.65606121 | 1.383465378 |
| Inverse variance weighted | 25 | 0.007458757 | 0.13224259 | 0.955021501 | 1.007486643 | 0.777449399 | 1.305588939 |
| Simple mode | 25 | -0.404634685 | 0.36216274 | 0.274937962 | 0.667220512 | 0.328087921 | 1.356902171 |
| Weighted mode | 25 | -0.2510135 | 0.304531174 | 0.417907461 | 0.778011869 | 0.428315759 | 1.413215497 |
| **Duodenal ulcer** | **SNPs** | **Beta** | **SE** | ***p* value** | **or** | **or_lci95** | **or_uci95** |
| MR Egger | 25 | -0.001317001 | 0.001683785 | 0.442094908 | 0.998683866 | 0.995393424 | 1.001985185 |
| Weighted median | 25 | -0.001225127 | 0.000799594 | 0.125476328 | 0.998775623 | 0.997211565 | 1.000342135 |
| Inverse variance weighted | 25 | -0.001170736 | 0.000577725 | **0.042717869** | 0.998829949 | 0.997699573 | 0.999961606 |
| Simple mode | 25 | -0.001260242 | 0.00137723 | 0.369265094 | 0.998740552 | 0.996048216 | 1.001440164 |
| Weighted mode | 25 | -0.00108783 | 0.001457724 | 0.462762738 | 0.998912762 | 0.996062802 | 1.001770875 |
| **Gastric ulcer** | **SNPs** | **Beta** | **SE** | ***p* value** | **or** | **or_lci95** | **or_uci95** |
| MR Egger | 25 | -0.055240792 | 0.167236318 | 0.744151359 | 0.94625727 | 0.681796553 | 1.31329913 |
| Weighted median | 25 | -0.001372305 | 0.07504794 | 0.985410926 | 0.998628636 | 0.862029085 | 1.156874136 |
| Inverse variance weighted | 25 | 0.030634933 | 0.056708616 | 0.589047705 | 1.031109011 | 0.922642028 | 1.152327511 |
| Simple mode | 25 | 0.02149534 | 0.146484623 | 0.884561931 | 1.021728029 | 0.766734631 | 1.361524736 |
| Weighted mode | 25 | -0.013421801 | 0.103331941 | 0.897735868 | 0.98666787 | 0.805773652 | 1.2081724 |
| **Acute gastritis** | **SNPs** | **Beta** | **SE** | ***p* value** | **or** | **or_lci95** | **or_uci95** |
| MR Egger | 22 | 0.161276751 | 0.455622968 | 0.727065911 | 1.175010109 | 0.481069146 | 2.869959067 |
| Weighted median | 22 | -0.012944014 | 0.244895407 | 0.957847219 | 0.987139399 | 0.610828521 | 1.595282734 |
| Inverse variance weighted | 22 | 0.129388959 | 0.169984875 | 0.446550013 | 1.138132725 | 0.815640654 | 1.588133321 |
| Simple mode | 22 | -0.320219508 | 0.446866018 | 0.481521611 | 0.725989659 | 0.302378147 | 1.7430525 |
| Weighted mode | 22 | -0.2802119 | 0.470745364 | 0.558036087 | 0.755623608 | 0.300330179 | 1.901131078 |
| **Chronic gastritis** | **SNPs** | **Beta** | **SE** | ***p* value** | **or** | **or_lci95** | **or_uci95** |
| MR Egger | 25 | -0.298993776 | 0.290397605 | 0.313903617 | 0.741564025 | 0.419717639 | 1.31020751 |
| Weighted median | 25 | 0.009373357 | 0.142492653 | 0.947551918 | 1.009417424 | 0.763446489 | 1.334636482 |
| Inverse variance weighted | 25 | 0.066166835 | 0.10817319 | 0.540752815 | 1.068404949 | 0.864285073 | 1.320732211 |
| Simple mode | 25 | -0.004278407 | 0.268139228 | 0.987401465 | 0.995730732 | 0.588704279 | 1.684172727 |
| Weighted mode | 25 | -0.030728686 | 0.250945073 | 0.903560795 | 0.969738641 | 0.592988052 | 1.58585494 |
| **Gastric cancer** | **SNPs** | **Beta** | **SE** | ***p* value** | **or** | **or_lci95** | **or_uci95** |
| MR Egger | 25 | -0.522006973 | 0.245686343 | **0.044576395** | 0.593328558 | 0.366574977 | 0.960345905 |
| Weighted median | 25 | -0.133140995 | 0.122258594 | 0.276148886 | 0.875341664 | 0.688825132 | 1.112362186 |
| Inverse variance weighted | 25 | -0.141828141 | 0.081155265 | 0.080530752 | 0.867770378 | 0.740157365 | 1.017385578 |
| Simple mode | 25 | 0.058459941 | 0.197744938 | 0.770053038 | 1.060202515 | 0.719556566 | 1.56211398 |
| Weighted mode | 25 | -0.108898236 | 0.167059872 | 0.520691172 | 0.896821677 | 0.646400767 | 1.244257683 |
| **Irritable bowel syndrome** | **SNPs** | **Beta** | **SE** | ***p* value** | **or** | **or_lci95** | **or_uci95** |
| MR Egger | 25 | 0.000983931 | 0.092877342 | 0.991638824 | 1.000984415 | 0.834388916 | 1.200842653 |
| Weighted median | 25 | 0.017699803 | 0.038610216 | 0.646648714 | 1.017857373 | 0.943672388 | 1.097874268 |
| Inverse variance weighted | 25 | 0.024245915 | 0.032113997 | 0.45025183 | 1.024542238 | 0.962041665 | 1.09110326 |
| Simple mode | 25 | 0.056866056 | 0.069053894 | 0.418330683 | 1.05851402 | 0.924520943 | 1.211927041 |
| Weighted mode | 25 | 0.044667149 | 0.054458703 | 0.420172729 | 1.045679747 | 0.939815304 | 1.163469171 |
| **Celiac disease** | **SNPs** | **Beta** | **SE** | ***p* value** | **or** | **or_lci95** | **or_uci95** |
| MR Egger | 3 | 1.51478688 | 0.731126136 | 0.286274637 | 4.548451659 | 1.085216659 | 19.0638545 |
| Weighted median | 3 | 0.105246722 | 0.310262536 | 0.734445305 | 1.110984681 | 0.604793713 | 2.040839603 |
| Inverse variance weighted | 3 | 0.072763522 | 0.403063681 | 0.856739551 | 1.07547618 | 0.488096917 | 2.369711782 |
| Simple mode | 3 | 0.081166829 | 0.469867507 | 0.878752664 | 1.084551816 | 0.431808323 | 2.724015674 |
| Weighted mode | 3 | 0.092526414 | 0.376995044 | 0.829009673 | 1.096942116 | 0.523936978 | 2.296615919 |
| **Crohn's disease** | **SNPs** | **Beta** | **SE** | ***p* value** | **or** | **or_lci95** | **or_uci95** |
| MR Egger | 18 | 1.421994321 | 1.342436579 | 0.305209752 | 4.14537942 | 0.298441491 | 57.57969664 |
| Weighted median | 18 | 1.568997956 | 0.653922674 | **0.016423618** | 4.801834134 | 1.332836684 | 17.29965218 |
| Inverse variance weighted | 18 | 1.021042149 | 0.493420971 | **0.038516778** | 2.776086352 | 1.055418157 | 7.301992468 |
| Simple mode | 18 | 1.777457832 | 1.007655025 | 0.095700677 | 5.914800873 | 0.820742372 | 42.62588429 |
| Weighted mode | 18 | 1.816972567 | 0.819558172 | **0.040542815** | 6.153201818 | 1.234466077 | 30.67066266 |
| **Ulcerative Colitis** | **SNPs** | **Beta** | **SE** | ***p* value** | **or** | **or_lci95** | **or_uci95** |
| MR Egger | 25 | 0.152090014 | 0.46030891 | 0.744082146 | 1.164265031 | 0.472312021 | 2.869952496 |
| Weighted median | 25 | -0.059725934 | 0.111337642 | 0.591654687 | 0.942022675 | 0.75733642 | 1.171747056 |
| Inverse variance weighted | 25 | -0.136000416 | 0.163030634 | 0.404167387 | 0.872842269 | 0.634105168 | 1.201462573 |
| Simple mode | 25 | 0.037960858 | 0.188447704 | 0.842052312 | 1.038690576 | 0.717920355 | 1.502782454 |
| Weighted mode | 25 | -0.103728051 | 0.167242822 | 0.540955225 | 0.901470418 | 0.649518486 | 1.251155946 |
| **Colorectal Cancer** | **SNPs** | **Beta** | **SE** | ***p* value** | **or** | **or_lci95** | **or_uci95** |
| MR Egger | 25 | -0.107920748 | 0.190366331 | 0.576266173 | 0.897698738 | 0.61814096 | 1.303688117 |
| Weighted median | 25 | -0.0101141 | 0.079715159 | 0.899036962 | 0.989936876 | 0.8467449 | 1.157343869 |
| Inverse variance weighted | 25 | -0.001248189 | 0.065016622 | 0.984683149 | 0.998752589 | 0.879254607 | 1.134491337 |
| Simple mode | 25 | -0.000405601 | 0.159561326 | 0.99799281 | 0.999594481 | 0.731143304 | 1.366611882 |
| Weighted mode | 25 | -0.028849625 | 0.117934453 | 0.80882677 | 0.971562552 | 0.771050697 | 1.224217546 |
| **Diverticular disease** | **SNPs** | **Beta** | **SE** | ***p* value** | **or** | **or_lci95** | **or_uci95** |
| MR Egger | 22 | -8.05E-05 | 0.003349012 | 0.981071765 | 0.999919547 | 0.993377506 | 1.006504673 |
| Weighted median | 22 | 0.000808746 | 0.001592667 | 0.611598301 | 1.000809073 | 0.997689791 | 1.003938107 |
| Inverse variance weighted | 22 | 0.000266877 | 0.001135614 | 0.814203372 | 1.000266913 | 0.998042992 | 1.002495789 |
| Simple mode | 22 | 0.001915611 | 0.003390232 | 0.578034678 | 1.001917447 | 0.995281921 | 1.008597213 |
| Weighted mode | 22 | 0.00208547 | 0.003971892 | 0.605043205 | 1.002087646 | 0.994316772 | 1.009919252 |
| **Acute pancreatitis** | **SNPs** | **Beta** | **SE** | ***p* value** | **or** | **or_lci95** | **or_uci95** |
| MR Egger | 25 | -0.118093232 | 0.242447213 | 0.630805644 | 0.888613201 | 0.552506672 | 1.429183504 |
| Weighted median | 25 | 0.087672211 | 0.119630278 | 0.463643996 | 1.091630239 | 0.863463948 | 1.3800884 |
| Inverse variance weighted | 25 | 0.041402235 | 0.086699587 | 0.632979991 | 1.042271259 | 0.879388099 | 1.235324176 |
| Simple mode | 25 | 0.132296684 | 0.207321647 | 0.529436002 | 1.141446919 | 0.760291214 | 1.713686866 |
| Weighted mode | 25 | 0.111987753 | 0.201086874 | 0.582744081 | 1.118499162 | 0.754166193 | 1.65883911 |
| **Chronic Pancreatitis** | **SNPs** | **Beta** | **SE** | ***p* value** | **or** | **or_lci95** | **or_uci95** |
| MR Egger | 25 | -0.607273881 | 0.390915626 | 0.133965236 | 0.544834129 | 0.253227114 | 1.172245037 |
| Weighted median | 25 | 0.02544496 | 0.20155619 | 0.899539961 | 1.025771446 | 0.691007105 | 1.522715255 |
| Inverse variance weighted | 25 | 0.05743724 | 0.140620705 | 0.682939611 | 1.059118798 | 0.803981241 | 1.395222389 |
| Simple mode | 25 | 0.366132018 | 0.411138597 | 0.382021032 | 1.442145619 | 0.644229898 | 3.228325778 |
| Weighted mode | 25 | 0.293172099 | 0.368164639 | 0.433660255 | 1.340673499 | 0.651530809 | 2.758742036 |
| **Pancreatic Cancer** | **SNPs** | **Beta** | **SE** | ***p* value** | **or** | **or_lci95** | **or_uci95** |
| MR Egger | 25 | 0.508469487 | 0.408094389 | 0.225321639 | 1.662744394 | 0.747220082 | 3.700006176 |
| Weighted median | 25 | 0.33910556 | 0.214168947 | 0.113340482 | 1.403691511 | 0.922502146 | 2.135875637 |
| Inverse variance weighted | 25 | 0.381413405 | 0.14409055 | **0.008119982** | 1.464352851 | 1.104061802 | 1.942218513 |
| Simple mode | 25 | 0.326546992 | 0.381712155 | 0.400745833 | 1.386173387 | 0.655990592 | 2.929122282 |
| Weighted mode | 25 | 0.433689607 | 0.328995707 | 0.199872508 | 1.542939877 | 0.809659105 | 2.940328157 |
| **Cholelithiasis** | **SNPs** | **Beta** | **SE** | ***p* value** | **or** | **or_lci95** | **or_uci95** |
| MR Egger | 25 | -0.108215916 | 0.119393151 | 0.374131401 | 0.897433805 | 0.710187336 | 1.134049278 |
| Weighted median | 25 | 0.056099473 | 0.051017106 | 0.271497359 | 1.057702891 | 0.957055348 | 1.168934909 |
| Inverse variance weighted | 25 | 0.073798948 | 0.043822385 | 0.092172622 | 1.076590333 | 0.987979899 | 1.173148104 |
| Simple mode | 25 | 0.063948613 | 0.087301614 | 0.470956019 | 1.066037616 | 0.898379629 | 1.264984382 |
| Weighted mode | 25 | 0.055221668 | 0.071507485 | 0.447504684 | 1.056774842 | 0.918573826 | 1.21576844 |
| **Cholecystitis** | **SNPs** | **Beta** | **SE** | ***p* value** | **or** | **or_lci95** | **or_uci95** |
| MR Egger | 25 | -0.11415191 | 0.193637028 | 0.561262763 | 0.892122423 | 0.610375776 | 1.303922025 |
| Weighted median | 25 | 0.02878396 | 0.07970147 | 0.717989439 | 1.029202221 | 0.880354227 | 1.203217047 |
| Inverse variance weighted | 25 | 0.095263766 | 0.068565487 | 0.164716121 | 1.099948946 | 0.961630894 | 1.258162245 |
| Simple mode | 25 | 0.029474837 | 0.152803292 | 0.848665038 | 1.029913519 | 0.76336452 | 1.389535182 |
| Weighted mode | 25 | 0.002411608 | 0.114151429 | 0.983319494 | 1.002414518 | 0.801456011 | 1.25376172 |
| **Nonalcoholic fatty liver disease** | **SNPs** | **Beta** | **SE** | **p value** | **or** | **or_lci95** | **or_uci95** |
| MR Egger | 17 | -0.032211149 | 0.227850705 | 0.889459156 | 0.968302104 | 0.619527287 | 1.51342642 |
| Weighted median | 17 | 0.05482909 | 0.109623673 | 0.616964262 | 1.056360056 | 0.852115405 | 1.309560374 |
| Inverse variance weighted | 17 | -0.035132271 | 0.088090127 | 0.690024442 | 0.965477703 | 0.812378496 | 1.147429676 |
| Simple mode | 17 | 0.171667145 | 0.182100037 | 0.359848085 | 1.187282574 | 0.830897424 | 1.696526996 |
| Weighted mode | 17 | 0.070200192 | 0.14249089 | 0.628941998 | 1.072722911 | 0.811328743 | 1.41833314 |
| **Alcoholic liver disease** | **SNPs** | **Beta** | **SE** | ***p* value** | **or** | **or_lci95** | **or_uci95** |
| MR Egger | 22 | -0.157811164 | 0.433583556 | 0.719700245 | 0.854011035 | 0.365081401 | 1.997732136 |
| Weighted median | 22 | -0.203501538 | 0.226537363 | 0.369018516 | 0.81586895 | 0.523344795 | 1.271899806 |
| Inverse variance weighted | 22 | -0.106373922 | 0.162093447 | 0.511662895 | 0.899088396 | 0.654373442 | 1.235318997 |
| Simple mode | 22 | -0.220905619 | 0.375574522 | 0.562684018 | 0.80179235 | 0.384031122 | 1.674007485 |
| Weighted mode | 22 | -0.202820191 | 0.340466847 | 0.557734101 | 0.816425029 | 0.418894906 | 1.591210154 |
| **Cirrhosis** | **SNPs** | **Beta** | **SE** | ***p* value** | **or** | **or_lci95** | **or_uci95** |
| MR Egger | 25 | 0.004290479 | 0.458314658 | 0.992611499 | 1.004299696 | 0.409013853 | 2.465974858 |
| Weighted median | 25 | -0.045547585 | 0.210209997 | 0.828460396 | 0.955474136 | 0.632826378 | 1.442624479 |
| Inverse variance weighted | 25 | -0.072895482 | 0.14815191 | 0.622696715 | 0.929697995 | 0.695396408 | 1.242943381 |
| Simple mode | 25 | 0.008178722 | 0.307918265 | 0.979029234 | 1.00821226 | 0.551374473 | 1.843560068 |
| Weighted mode | 25 | -0.021817194 | 0.224141896 | 0.92326747 | 0.97841908 | 0.630567349 | 1.518162805 |
| **Hepatic cancer** | **SNPs** | **Beta** | **SE** | ***p* value** | **or** | **or_lci95** | **or_uci95** |
| MR Egger | 25 | 1.384233071 | 0.582279649 | **0.026141268** | 3.991763333 | 1.275023946 | 12.49715706 |
| Weighted median | 25 | 0.373981502 | 0.222370621 | 0.092608858 | 1.453510263 | 0.940009859 | 2.247521199 |
| Inverse variance weighted | 25 | 0.329475752 | 0.204148134 | 0.106547972 | 1.390239108 | 0.931783684 | 2.074263383 |
| Simple mode | 25 | 0.155088481 | 0.347954068 | 0.659797751 | 1.167761282 | 0.590431595 | 2.309609482 |
| Weighted mode | 25 | 0.205400444 | 0.268751933 | 0.452152557 | 1.228016719 | 0.725166973 | 2.079555632 |
| **Acute appendicitis** | **SNPs** | **Beta** | **SE** | ***p* value** | **or** | **or_lci95** | **or_uci95** |
| MR Egger | 22 | -0.118110137 | 0.13896392 | 0.405421965 | 0.88859818 | 0.676732357 | 1.166793219 |
| Weighted median | 22 | -0.064708417 | 0.069869514 | 0.354377206 | 0.937340736 | 0.817378783 | 1.074908811 |
| Inverse variance weighted | 22 | -0.020226946 | 0.051913148 | 0.696809801 | 0.979976247 | 0.885168978 | 1.084937981 |
| Simple mode | 22 | -0.054854022 | 0.116706544 | 0.643187448 | 0.946623324 | 0.75306868 | 1.189925623 |
| Weighted mode | 22 | -0.092591129 | 0.0992414 | 0.361429642 | 0.911566137 | 0.750433475 | 1.107297116 |

Beta, regression coefficient. SE, Standard Error. or, odds ratio. or_lci95, lower confidence interval of 95% for the odds ratio. or_uci95, upper confidence agreement of 95% for the odds ratio.

**Table S8 Horizontal pleiotropy test**

| **Outcome** | **Exposure** | **Egger_intercept** | **SE** | ***P* value** |
| --- | --- | --- | --- | --- |
| Gastroesophageal reflux disease | Cataracts | 0.001042 | 0.02179 | 0.963706 |
| Esophageal cancer | Cataracts | 0.048857 | 0.023516 | 0.049097 |
| Duodenal ulcer | Cataracts | 1.03E-05 | 0.000111 | 0.926907 |
| Gastric ulcer | Cataracts | 0.005532 | 0.010116 | 0.589709 |
| Acute gastritis | Cataracts | -0.00215 | 0.028526 | 0.940619 |
| Chronic gastritis | Cataracts | 0.024524 | 0.018147 | 0.189728 |
| Gastric cancer | Cataracts | 0.023351 | 0.014243 | 0.11473 |
| Irritable bowel syndrome | Cataracts | 0.001624 | 0.006068 | 0.79135 |
| Celiac disease | Cataracts | -0.08022 | 0.037932 | 0.281197 |
| Crohn's disease | Cataracts | -0.02709 | 0.083977 | 0.751205 |
| Ulcerative colitis | Cataracts | -0.01987 | 0.029636 | 0.509295 |
| Colorectal cancer | Cataracts | 0.007014 | 0.011744 | 0.556148 |
| diverticular disease | Cataracts | 2.36E-05 | 0.000214 | 0.913037 |
| Acute pancreatitis | Cataracts | 0.010853 | 0.015406 | 0.488232 |
| Chronic pancreatitis | Cataracts | 0.04468 | 0.024517 | 0.081425 |
| Pancreatic cancer | Cataracts | -0.00859 | 0.0258 | 0.74232 |
| Cholelithiasis | Cataracts | 0.012278 | 0.00753 | 0.116573 |
| Cholecystitis | Cataracts | 0.014403 | 0.012467 | 0.259841 |
| Nonalcoholic fatty liver disease | Cataracts | -0.0002 | 0.01457 | 0.989028 |
| Alcoholic liver disease | Cataracts | 0.003474 | 0.027162 | 0.8995 |
| Cirrhosis | Cataracts | -0.00466 | 0.026164 | 0.860307 |
| Hepatic cancer | Cataracts | -0.06516 | 0.033927 | 0.067253 |
| Acute appendicitis | Cataracts | 0.00661 | 0.008705 | 0.456498 |

**Table S9 Heterogeneity test of the association between cataracts with 23 gastrointestinal diseases**

| **Exposure** | **Outcome** | **Method** | **Q** | **Q_df** | **Q_pval** |
| --- | --- | --- | --- | --- | --- |
| Cataracts | Gastroesophageal reflux disease | MR Egger | 29.33811767 | 5 | **1.99E-05** |
| Cataracts | Gastroesophageal reflux disease | Inverse variance weighted | 29.35153885 | 6 | **5.22E-05** |
| Cataracts | Esophageal Cancer | MR Egger | 19.75100983 | 23 | 0.656881459 |
| Cataracts | Esophageal Cancer | Inverse variance weighted | 24.06741044 | 24 | 0.457748001 |
| Cataracts | Duodenal ulcer | MR Egger | 27.81119437 | 23 | 0.223005667 |
| Cataracts | Duodenal ulcer | Inverse variance weighted | 27.821596 | 24 | 0.267636697 |
| Cataracts | Gastric ulcer | MR Egger | 30.24117976 | 23 | 0.142602642 |
| Cataracts | Gastric ulcer | Inverse variance weighted | 30.63445812 | 24 | 0.164604397 |
| Cataracts | Acute gastritis | MR Egger | 17.15066117 | 20 | 0.643170023 |
| Cataracts | Acute gastritis | Inverse variance weighted | 17.15635141 | 21 | 0.701585851 |
| Cataracts | Chronic gastritis | MR Egger | 29.34206132 | 23 | 0.169235109 |
| Cataracts | Chronic gastritis | Inverse variance weighted | 31.67174218 | 24 | 0.135343648 |
| Cataracts | Gastric cancer | MR Egger | 17.59458365 | 23 | 0.779093584 |
| Cataracts | Gastric cancer | Inverse variance weighted | 20.28234425 | 24 | 0.680611752 |
| Cataracts | Irritable bowel syndrome | MR Egger | 35.68350271 | 23 | **0.044424419** |
| Cataracts | Irritable bowel syndrome | Inverse variance weighted | 35.79464799 | 24 | 0.057455947 |
| Cataracts | Celiac disease | MR Egger | 0.197131747 | 1 | 0.657046046 |
| Cataracts | Celiac disease | Inverse variance weighted | 4.669407623 | 2 | 0.096839161 |
| Cataracts | Crohn's disease | MR Egger | 19.12274797 | 16 | 0.262349277 |
| Cataracts | Crohn's disease | Inverse variance weighted | 19.24709959 | 17 | 0.31453465 |
| Cataracts | Ulcerative Colitis | MR Egger | 108.8347725 | 23 | **4.05E-13** |
| Cataracts | Ulcerative Colitis | Inverse variance weighted | 110.9613184 | 24 | **3.83E-13** |
| Cataracts | Colorectal Cancer | MR Egger | 34.09656775 | 23 | 0.063774708 |
| Cataracts | Colorectal Cancer | Inverse variance weighted | 34.62544545 | 24 | 0.074151176 |
| Cataracts | Diverticular disease | MR Egger | 25.11663691 | 20 | 0.197005252 |
| Cataracts | Diverticular disease | Inverse variance weighted | 25.13199832 | 21 | 0.241492398 |
| Cataracts | Acute pancreatitis | MR Egger | 20.69368658 | 23 | 0.599787569 |
| Cataracts | Acute pancreatitis | Inverse variance weighted | 21.18991956 | 24 | 0.627497504 |
| Cataracts | Chronic Pancreatitis | MR Egger | 19.963372 | 23 | 0.644120362 |
| Cataracts | Chronic Pancreatitis | Inverse variance weighted | 23.28446394 | 24 | 0.503061456 |
| Cataracts | Pancreatic Cancer | MR Egger | 21.50989548 | 23 | 0.54997907 |
| Cataracts | Pancreatic Cancer | Inverse variance weighted | 21.62063334 | 24 | 0.601898345 |
| Cataracts | Cholelithiasis | MR Egger | 35.39844309 | 23 | **0.047462838** |
| Cataracts | Cholelithiasis | Inverse variance weighted | 39.49108984 | 24 | **0.024236947** |
| Cataracts | Cholecystitis | MR Egger | 38.09229655 | 23 | **0.024897077** |
| Cataracts | Cholecystitis | Inverse variance weighted | 40.30272775 | 24 | **0.01983959** |
| Cataracts | Nonalcoholic fatty liver disease | MR Egger | 21.08794213 | 15 | 0.134042993 |
| Cataracts | Nonalcoholic fatty liver disease | Inverse variance weighted | 21.08821702 | 16 | 0.175144473 |
| Cataracts | Alcoholic liver disease | MR Egger | 16.6281213 | 20 | 0.676972394 |
| Cataracts | Alcoholic liver disease | Inverse variance weighted | 16.64448157 | 21 | 0.732418807 |
| Cataracts | Cirrhosis | MR Egger | 15.26844678 | 23 | 0.884754809 |
| Cataracts | Cirrhosis | Inverse variance weighted | 15.30011912 | 24 | 0.911669038 |
| Cataracts | Hepatic cancer | MR Egger | 39.84117199 | 23 | **0.016011033** |
| Cataracts | Hepatic cancer | Inverse variance weighted | 46.23142462 | 24 | **0.004151981** |
| Cataracts | Acute appendicitis | MR Egger | 16.16300768 | 20 | 0.706461402 |
| Cataracts | Acute appendicitis | Inverse variance weighted | 16.73962788 | 21 | 0.726763644 |

**Table S10.** **Multivariable Mendelian randomization results.**

| **Exposure** | **Method** | **or** | **or_lci95** | **or_uci95** | **pval** |
| --- | --- | --- | --- | --- | --- |
| Gastroesophageal reflux disease | mr_mvivw | 1.148567812 | 1.072099304 | 1.23049051 | **8.13E-05** |
| HDL cholesterol | mr_mvivw | 0.992354029 | 0.950719877 | 1.035811434 | 0.725594196 |
| Gastroesophageal reflux disease | mr_mvegger | 1.172620225 | 1.045850025 | 1.31475657 | **0.006372083** |
| HDL cholesterol | mr_mvegger | 0.991738874 | 0.949917236 | 1.035401778 | 0.705897296 |
| Gastroesophageal reflux disease | mr_mvlasso | 1.110983375 | 1.048505168 | 1.177184526 | **0.000365323** |
| HDL cholesterol | mr_mvlasso | 0.981137221 | 0.946260455 | 1.017299457 | 0.302441342 |
| Gastroesophageal reflux disease | mr_mvmedian | 1.130932374 | 1.041298867 | 1.228281405 | **0.003493747** |
| HDL cholesterol | mr_mvmedian | 0.99671193 | 0.94680677 | 1.049247537 | 0.899993539 |
| Gastroesophageal reflux disease | mr_mvivw | 1.096048433 | 1.024318158 | 1.172801789 | **0.007912535** |
| Total cholesterol | mr_mvivw | 0.988952567 | 0.953582572 | 1.025634494 | 0.549948139 |
| Gastroesophageal reflux disease | mr_mvegger | 1.069121957 | 0.958367002 | 1.192676457 | 0.230966837 |
| Total cholesterol | mr_mvegger | 0.989937264 | 0.954279343 | 1.02692759 | 0.588954614 |
| Gastroesophageal reflux disease | mr_mvlasso | 1.084259353 | 1.020597018 | 1.151892789 | **0.008782821** |
| Total cholesterol | mr_mvlasso | 0.99754104 | 0.965750326 | 1.030378246 | 0.881561131 |
| Gastroesophageal reflux disease | mr_mvmedian | 1.078985395 | 0.98788304 | 1.17848919 | 0.091195132 |
| Total cholesterol | mr_mvmedian | 0.992920534 | 0.945681739 | 1.042519006 | 0.775126992 |
| Gastroesophageal reflux disease | mr_mvivw | 1.101121468 | 1.035536333 | 1.170860402 | **0.002108431** |
| Triglycerides | mr_mvivw | 0.960571677 | 0.923185369 | 0.999472022 | **0.047025079** |
| Gastroesophageal reflux disease | mr_mvegger | 1.11886778 | 0.998556075 | 1.253675322 | 0.052977442 |
| Triglycerides | mr_mvegger | 0.959987257 | 0.922312677 | 0.999200767 | **0.04559287** |
| Gastroesophageal reflux disease | mr_mvlasso | 1.089325171 | 1.029234267 | 1.152924428 | **0.003123477** |
| Triglycerides | mr_mvlasso | 0.966985178 | 0.932333461 | 1.002924784 | 0.071366127 |
| Gastroesophageal reflux disease | mr_mvmedian | 1.084973824 | 0.999631071 | 1.177602651 | 0.051036592 |
| Triglycerides | mr_mvmedian | 0.969881837 | 0.918554329 | 1.024077453 | 0.270305275 |
| Celiac disease | mr_mvivw | 1.012295493 | 1.004145932 | 1.020511195 | **0.003044335** |
| LDL cholesterol | mr_mvivw | 1.001584954 | 0.952553931 | 1.053139761 | 0.950687694 |
| Celiac disease | mr_mvegger | 1.01578656 | 1.005031299 | 1.026656918 | **0.003925366** |
| LDL cholesterol | mr_mvegger | 1.008514708 | 0.957319267 | 1.062447975 | 0.749737609 |
| Celiac disease | mr_mvlasso | 1.012295493 | 1.004145932 | 1.020511195 | 0.003044335 |
| LDL cholesterol | mr_mvlasso | 1.001584954 | 0.952553931 | 1.053139761 | 0.950687694 |
| Celiac disease | mr_mvmedian | 1.005457255 | 0.992537331 | 1.018545358 | 0.409487718 |
| LDL cholesterol | mr_mvmedian | 0.986901202 | 0.916466638 | 1.062748977 | 0.72707082 |
| Celiac disease | mr_mvivw | 1.011627875 | 1.001998738 | 1.021349548 | **0.017826565** |
| HDL cholesterol | mr_mvivw | 1.016126055 | 0.954746261 | 1.081451901 | 0.614801059 |
| Celiac disease | mr_mvegger | 1.015403653 | 1.003029797 | 1.027930159 | **0.014541531** |
| HDL cholesterol | mr_mvegger | 1.021926348 | 0.959090793 | 1.08887862 | 0.502920406 |
| Celiac disease | mr_mvlasso | 1.00774543 | 0.999224033 | 1.016339499 | 0.074940238 |
| HDL cholesterol | mr_mvlasso | 1.013187502 | 0.96013018 | 1.0691768 | 0.633073261 |
| Celiac disease | mr_mvmedian | 1.005649315 | 0.99252081 | 1.018951476 | 0.400767772 |
| HDL cholesterol | mr_mvmedian | 0.982832569 | 0.912974193 | 1.058036325 | 0.645282567 |
| Celiac disease | mr_mvivw | 1.011789216 | 1.003715821 | 1.01992755 | **0.004138546** |
| Triglycerides | mr_mvivw | 0.990386325 | 0.940275417 | 1.043167837 | 0.715365381 |
| Celiac disease | mr_mvegger | 1.016783611 | 1.006196553 | 1.027482064 | **0.00182845** |
| Triglycerides | mr_mvegger | 0.988432558 | 0.938354609 | 1.041183059 | 0.660945401 |
| Celiac disease | mr_mvlasso | 1.011789216 | 1.003715821 | 1.01992755 | **0.004138546** |
| Triglycerides | mr_mvlasso | 0.990386325 | 0.940275417 | 1.043167837 | 0.715365381 |
| Celiac disease | mr_mvmedian | 1.005504561 | 0.992530736 | 1.018647972 | 0.407395303 |
| Triglycerides | mr_mvmedian | 0.981509315 | 0.91372156 | 1.05432615 | 0.609243744 |

**Table S11. Identifying 67 plasma metabolites with potential causal effects on cataract risk through two-step MR analysis.**

| **outcome** | **exposure** | **nsnp** | **or** | **or_lci95** | **or_uci95** | **pval** |
| --- | --- | --- | --- | --- | --- | --- |
| Cataracts \|\| id:ebi-a-GCST90018814 | GCST90199623 | 22 | 1.039407435 | 1.004547623 | 1.075476952 | 0.026371774 |
| Cataracts \|\| id:ebi-a-GCST90018814 | GCST90199651 | 20 | 1.047367483 | 1.002022091 | 1.09476493 | 0.040417567 |
| Cataracts \|\| id:ebi-a-GCST90018814 | GCST90199653 | 17 | 0.945118056 | 0.900972687 | 0.991426435 | 0.020733082 |
| Cataracts \|\| id:ebi-a-GCST90018814 | GCST90199660 | 26 | 1.029963414 | 1.000903644 | 1.059866891 | 0.043191152 |
| Cataracts \|\| id:ebi-a-GCST90018814 | GCST90199675 | 23 | 1.035587107 | 1.008728538 | 1.063160817 | 0.009101573 |
| Cataracts \|\| id:ebi-a-GCST90018814 | GCST90199676 | 26 | 1.041912577 | 1.003814136 | 1.081456994 | 0.030749289 |
| Cataracts \|\| id:ebi-a-GCST90018814 | GCST90199721 | 23 | 1.042214787 | 1.003562849 | 1.082355395 | 0.031996585 |
| Cataracts \|\| id:ebi-a-GCST90018814 | GCST90199722 | 25 | 0.969540586 | 0.940247073 | 0.999746742 | 0.048134204 |
| Cataracts \|\| id:ebi-a-GCST90018814 | GCST90199738 | 19 | 1.056425699 | 1.010734531 | 1.104182377 | 0.014960854 |
| Cataracts \|\| id:ebi-a-GCST90018814 | GCST90199754 | 22 | 1.048832947 | 1.004470284 | 1.095154898 | 0.03059634 |
| Cataracts \|\| id:ebi-a-GCST90018814 | GCST90199765 | 16 | 0.929264119 | 0.882649774 | 0.978340253 | 0.005206608 |
| Cataracts \|\| id:ebi-a-GCST90018814 | GCST90199793 | 30 | 0.952220453 | 0.928143492 | 0.976921994 | 0.000179031 |
| Cataracts \|\| id:ebi-a-GCST90018814 | GCST90199794 | 25 | 0.957396054 | 0.920614093 | 0.995647591 | 0.02938908 |
| Cataracts \|\| id:ebi-a-GCST90018814 | GCST90199857 | 21 | 1.028056694 | 1.002648256 | 1.054109015 | 0.030224403 |
| Cataracts \|\| id:ebi-a-GCST90018814 | GCST90199865 | 24 | 1.061060594 | 1.024768274 | 1.098638212 | 0.000844136 |
| Cataracts \|\| id:ebi-a-GCST90018814 | GCST90199868 | 21 | 0.971190067 | 0.947293728 | 0.995689212 | 0.021455055 |
| Cataracts \|\| id:ebi-a-GCST90018814 | GCST90199876 | 30 | 1.031834267 | 1.003149756 | 1.061338995 | 0.029359352 |
| Cataracts \|\| id:ebi-a-GCST90018814 | GCST90199877 | 12 | 1.028929662 | 1.002367271 | 1.056195947 | 0.032582447 |
| Cataracts \|\| id:ebi-a-GCST90018814 | GCST90199933 | 24 | 1.035470022 | 1.000472024 | 1.071692302 | 0.046933375 |
| Cataracts \|\| id:ebi-a-GCST90018814 | GCST90199935 | 16 | 0.95771009 | 0.918703388 | 0.998372956 | 0.041674619 |
| Cataracts \|\| id:ebi-a-GCST90018814 | GCST90199948 | 17 | 0.966924149 | 0.937164782 | 0.997628515 | 0.034956312 |
| Cataracts \|\| id:ebi-a-GCST90018814 | GCST90199967 | 18 | 0.949454986 | 0.91521322 | 0.984977873 | 0.00564577 |
| Cataracts \|\| id:ebi-a-GCST90018814 | GCST90199969 | 24 | 0.971754676 | 0.948917653 | 0.995141303 | 0.018205218 |
| Cataracts \|\| id:ebi-a-GCST90018814 | GCST90199992 | 23 | 0.967915714 | 0.94580377 | 0.990544613 | 0.005679256 |
| Cataracts \|\| id:ebi-a-GCST90018814 | GCST90200126 | 21 | 1.056449865 | 1.000609393 | 1.115406596 | 0.047480604 |
| Cataracts \|\| id:ebi-a-GCST90018814 | GCST90200130 | 17 | 0.940237216 | 0.893196807 | 0.989755019 | 0.018610044 |
| Cataracts \|\| id:ebi-a-GCST90018814 | GCST90200173 | 14 | 1.098010835 | 1.040779728 | 1.158389004 | 0.000618189 |
| Cataracts \|\| id:ebi-a-GCST90018814 | GCST90200191 | 22 | 0.949430854 | 0.914130382 | 0.986094506 | 0.007266649 |
| Cataracts \|\| id:ebi-a-GCST90018814 | GCST90200195 | 33 | 1.037320484 | 1.006107895 | 1.069501384 | 0.01874053 |
| Cataracts \|\| id:ebi-a-GCST90018814 | GCST90200214 | 27 | 0.966902011 | 0.936399738 | 0.998397866 | 0.039586119 |
| Cataracts \|\| id:ebi-a-GCST90018814 | GCST90200219 | 18 | 1.029925642 | 1.002168003 | 1.0584521 | 0.034398955 |
| Cataracts \|\| id:ebi-a-GCST90018814 | GCST90200239 | 26 | 0.95787691 | 0.93026213 | 0.986311434 | 0.003932878 |
| Cataracts \|\| id:ebi-a-GCST90018814 | GCST90200344 | 17 | 0.93508894 | 0.892587739 | 0.979613866 | 0.004686146 |
| Cataracts \|\| id:ebi-a-GCST90018814 | GCST90200355 | 24 | 1.036351105 | 1.008154445 | 1.065336386 | 0.01117887 |
| Cataracts \|\| id:ebi-a-GCST90018814 | GCST90200389 | 17 | 0.936300507 | 0.887996745 | 0.987231815 | 0.014870987 |
| Cataracts \|\| id:ebi-a-GCST90018814 | GCST90200390 | 16 | 0.949576778 | 0.902362704 | 0.999261221 | 0.046767149 |
| Cataracts \|\| id:ebi-a-GCST90018814 | GCST90200442 | 19 | 0.958508814 | 0.918768503 | 0.999968048 | 0.049823176 |
| Cataracts \|\| id:ebi-a-GCST90018814 | GCST90200476 | 21 | 0.959041088 | 0.927598723 | 0.991549239 | 0.013932619 |
| Cataracts \|\| id:ebi-a-GCST90018814 | GCST90200519 | 23 | 0.961260815 | 0.92837291 | 0.995313786 | 0.026117291 |
| Cataracts \|\| id:ebi-a-GCST90018814 | GCST90200538 | 17 | 1.044999456 | 1.000561107 | 1.091411464 | 0.047111002 |
| Cataracts \|\| id:ebi-a-GCST90018814 | GCST90200545 | 17 | 1.05534159 | 1.014521645 | 1.097803951 | 0.007443065 |
| Cataracts \|\| id:ebi-a-GCST90018814 | GCST90200558 | 22 | 0.957999232 | 0.92356459 | 0.993717753 | 0.02159425 |
| Cataracts \|\| id:ebi-a-GCST90018814 | GCST90200568 | 21 | 1.038973905 | 1.006857485 | 1.072114765 | 0.017005333 |
| Cataracts \|\| id:ebi-a-GCST90018814 | GCST90200577 | 20 | 0.972525804 | 0.947407633 | 0.998309922 | 0.036915344 |
| Cataracts \|\| id:ebi-a-GCST90018814 | GCST90200592 | 24 | 0.971577509 | 0.946766808 | 0.997038393 | 0.028908662 |
| Cataracts \|\| id:ebi-a-GCST90018814 | GCST90200611 | 16 | 0.933966848 | 0.879975731 | 0.991270602 | 0.024538403 |
| Cataracts \|\| id:ebi-a-GCST90018814 | GCST90200614 | 23 | 0.955422578 | 0.915959404 | 0.996585983 | 0.034098414 |
| Cataracts \|\| id:ebi-a-GCST90018814 | GCST90200639 | 15 | 0.958934083 | 0.920700238 | 0.998755661 | 0.043385544 |
| Cataracts \|\| id:ebi-a-GCST90018814 | GCST90200642 | 22 | 0.981984808 | 0.967126003 | 0.997071902 | 0.019441385 |
| Cataracts \|\| id:ebi-a-GCST90018814 | GCST90200649 | 29 | 0.970384879 | 0.945527271 | 0.995895987 | 0.023170425 |
| Cataracts \|\| id:ebi-a-GCST90018814 | GCST90200665 | 23 | 0.965084607 | 0.937513634 | 0.993466405 | 0.016249313 |
| Cataracts \|\| id:ebi-a-GCST90018814 | GCST90200734 | 16 | 1.085816187 | 1.018352721 | 1.157748948 | 0.011879879 |
| Cataracts \|\| id:ebi-a-GCST90018814 | GCST90200745 | 22 | 0.957013956 | 0.919723057 | 0.995816845 | 0.030256178 |
| Cataracts \|\| id:ebi-a-GCST90018814 | GCST90200746 | 21 | 0.968467993 | 0.93863794 | 0.99924605 | 0.044722866 |
| Cataracts \|\| id:ebi-a-GCST90018814 | GCST90200754 | 15 | 0.93581609 | 0.883085336 | 0.991695501 | 0.024972977 |
| Cataracts \|\| id:ebi-a-GCST90018814 | GCST90200762 | 17 | 0.950508768 | 0.90585069 | 0.997368472 | 0.038703925 |
| Cataracts \|\| id:ebi-a-GCST90018814 | GCST90200796 | 21 | 1.07673998 | 1.025928719 | 1.130067776 | 0.002718309 |
| Cataracts \|\| id:ebi-a-GCST90018814 | GCST90200799 | 22 | 0.965896939 | 0.941605223 | 0.990815336 | 0.007584517 |
| Cataracts \|\| id:ebi-a-GCST90018814 | GCST90200806 | 20 | 1.009672662 | 1.000733865 | 1.018691303 | 0.033863717 |
| Cataracts \|\| id:ebi-a-GCST90018814 | GCST90200820 | 21 | 1.032841613 | 1.002910098 | 1.063666425 | 0.031265885 |
| Cataracts \|\| id:ebi-a-GCST90018814 | GCST90200822 | 25 | 1.042524453 | 1.001982692 | 1.084706597 | 0.039601953 |
| Cataracts \|\| id:ebi-a-GCST90018814 | GCST90200845 | 16 | 1.058853204 | 1.012194813 | 1.107662371 | 0.012876264 |
| Cataracts \|\| id:ebi-a-GCST90018814 | GCST90200854 | 21 | 1.048202455 | 1.011078917 | 1.086689049 | 0.01050066 |
| Cataracts \|\| id:ebi-a-GCST90018814 | GCST90200870 | 25 | 1.041167523 | 1.002389304 | 1.081445909 | 0.037230022 |
| Cataracts \|\| id:ebi-a-GCST90018814 | GCST90200944 | 12 | 0.93324742 | 0.889770295 | 0.978848982 | 0.004535566 |
| Cataracts \|\| id:ebi-a-GCST90018814 | GCST90200971 | 22 | 0.958976877 | 0.920046541 | 0.99955449 | 0.047582891 |
| Cataracts \|\| id:ebi-a-GCST90018814 | GCST90201006 | 15 | 1.052598844 | 1.006957184 | 1.100309272 | 0.023418033 |

**Table S12. Causal effects of gastroesophageal reflux disease on plasma metabolite levels through two-step MR analysis.**

| **exposure** | **nsnp** | **or** | **or_lci95** | **or_uci95** | **pval** |
| --- | --- | --- | --- | --- | --- |
| Gastroesophageal reflux disease \|\| id:ebi-a-GCST90000514 | 65 | 0.880311296 | 0.780845312 | 0.992447501 | 0.037166492 |
| Gastroesophageal reflux disease \|\| id:ebi-a-GCST90000514 | 65 | 1.137454616 | 1.006099496 | 1.285959299 | 0.039673971 |
| Gastroesophageal reflux disease \|\| id:ebi-a-GCST90000514 | 65 | 0.848560891 | 0.74789161 | 0.962780672 | 0.01081281 |
| Gastroesophageal reflux disease \|\| id:ebi-a-GCST90000514 | 65 | 0.832581494 | 0.736872558 | 0.940721618 | 0.003273641 |
| Gastroesophageal reflux disease \|\| id:ebi-a-GCST90000514 | 65 | 0.869644856 | 0.767030273 | 0.985987388 | 0.02923618 |
| Gastroesophageal reflux disease \|\| id:ebi-a-GCST90000514 | 65 | 1.199992316 | 1.064607779 | 1.352593496 | 0.002835224 |
| Gastroesophageal reflux disease \|\| id:ebi-a-GCST90000514 | 65 | 0.832867741 | 0.730834382 | 0.949146196 | 0.00609254 |
| Gastroesophageal reflux disease \|\| id:ebi-a-GCST90000514 | 65 | 1.15467428 | 1.016497253 | 1.31163433 | 0.026992711 |

**Table S13. Causal effects of celiac disease on plasma metabolite levels through two-step MR analysis.**

| **exposure** | **outcome** | **nsnp** | **or** | **or_lci95** | **or_uci95** | **pval** |
| --- | --- | --- | --- | --- | --- | --- |
| Celiac disease \|\| id:ebi-a-GCST005523 | Homoarginine levels | 38 | 1.085298906 | 1.050280272 | 1.121485138 | 1.00E-06 |
| Celiac disease \|\| id:ebi-a-GCST005523 | N-lactoyl tyrosine levels | 38 | 1.021496802 | 1.001088616 | 1.04232103 | 0.038859637 |
| Celiac disease \|\| id:ebi-a-GCST005523 | 11beta-hydroxyandrosterone glucuronide levels | 38 | 1.02028763 | 1.001728399 | 1.039190712 | 0.032002669 |
| Celiac disease \|\| id:ebi-a-GCST005523 | N6,n6,n6-trimethyllysine levels | 38 | 1.025269818 | 1.003837336 | 1.047159895 | 0.020594503 |
| Celiac disease \|\| id:ebi-a-GCST005523 | X-19438 levels | 38 | 1.036752976 | 1.016069447 | 1.057857548 | 0.000447259 |
| Celiac disease \|\| id:ebi-a-GCST005523 | X-21607 levels | 38 | 1.035556687 | 1.00174126 | 1.07051361 | 0.039140214 |
| Celiac disease \|\| id:ebi-a-GCST005523 | Alpha-ketoglutarate to pyruvate ratio | 38 | 0.979075337 | 0.95903353 | 0.999535977 | 0.0450718 |
